# Analysis of nonideality: insights from high concentration simulations of sedimentation velocity data

**DOI:** 10.1007/s00249-020-01474-5

**Published:** 2020-11-06

**Authors:** J. J. Correia, R. T. Wright, P. J. Sherwood, W. F. Stafford

**Affiliations:** 1 Department of Cell and Molecular Biology, University of MS Medical Center, Jackson, MS USA; 2 Biophysics Group, Janssen Biotherapeutics, Spring House, PA USA; 3 Interactive Technology, Oakland, CA USA; 4 Department of Systems Biology, Harvard Medical School, Boston, MA USA

**Keywords:** 2nd Virial coefficient, Diffusion, Hydrodynamic nonideality, Sedimentation velocity, Thermodynamic nonideality

## Abstract

The Aviv fluorescence detection system (Aviv-FDS) has allowed the performance of sedimentation velocity experiments on therapeutic antibodies in highly concentrated environments like formulation buffers and serum. Methods were implemented in the software package SEDANAL for the analysis of nonideal, weakly associating AUC data acquired on therapeutic antibodies and proteins (Wright et al. Eur Biophys J 47:709–722, 2018, Anal Biochem 550:72–83, 2018). This involved fitting both hydrodynamic, *k*_*s*_, and thermodynamic, BM_1_, nonideality where concentration dependence is expressed as *s* = *s*^o^/(1 + *k*_*s*_*c*) and *D* = *D*^o^(1 + 2BM_1_*c*)/(1 + *k*_*s*_*c*) and *s*^o^ and *D*^o^ are values extrapolated to *c* = 0 (mg/ml). To gain insight into the consequences of these phenomenological parameters, we performed simulations with SEDANAL of a monoclonal antibody as a function of *k*_*s*_ (0–100 ml/g) and BM_1_ (0–100 ml/g). This provides a visual understanding of the separate and joint impact of *k*_*s*_ and BM_1_ on the shape of high-concentration sedimentation velocity boundaries and the challenge of their unique determination by finite element methods. In addition, mAbs undergo weak self- and hetero-association (Yang et al. Prot Sci 27:1334–1348, 2018) and thus we have simulated examples of nonideal weak association over a wide range of concentrations (1–120 mg/ml). Here we demonstrate these data are best analyzed by direct boundary global fitting to models that account for *k*_*s*_, BM_1_ and weak association. Because a typical clinical dose of mAb is 50–200 mg/ml, these results have relevance for biophysical understanding of concentrated therapeutic proteins.

**Electronic supplementary material:**

The online version of this article (10.1007/s00249-020-01474-5) contains supplementary material, which is available to authorized users.

## Introduction

There is great interest in the biotechnology field in studying high concentration therapeutic monoclonal antibody (mAb) solutions. Monoclonal antibodies are typically administered by IV or subcutaneous (SC) injection at high concentrations (Rituxan: 10 mg/ml; Avastin: 25 mg/ml; Simponi: 100 mg/ml; Humira: 100 mg/ml; Herceptin: 150 mg/ml; Xolair: 150 mg/ml). This requires that mAbs be highly soluble and stable under formulation conditions. Analytical Ultracentrifugation (AUC) sedimentation velocity (SV) is highly appropriate for studying mAbs because it can reveal the presence of unfolding, dissociation, aggregation and undesirable reversible association over a wide range of concentrations (Berkowitz 2006; Philo 2009; Liu et al. 2005). To address the needs of therapeutic protein development, experiments at high concentration and in complex solution environments are required (Shire et al. 2004; Liu et al. 2015). The fluorescence detection system (FDS) developed by Tom Laue and colleagues (MacGregor et al. 2004; Kroe and Laue 2009; Kingsbury and Laue 2011) and, until recently, available from Aviv, is an extremely useful technique for studying high concentrations and heterogeneous systems like serum. FDS experiments with mAbs in serum can reveal unusual interactions that do not occur in typical formulation buffer conditions (Demeule et al. 2009). Nonideality is represented by the following phenomenological equations for sedimentation and diffusion (Fujita 1962).

1 $$s= \frac{{s}^{\mathrm{o}}}{1+{k}_{s}c} \,\text{and}$$

2 $$D= \frac{{D}^{\mathrm{o}} (1 + 2B{M}_{1}c )}{(1 + {k}_{s}c)}$$

In these equation *s*^o^ and *D*^o^ are values extrapolated to zero protein concentration, *c* is concentration in mg/ml, *k*_*s*_ is hydrodynamic nonideality and BM_1_ is thermodynamic nonideality (the 2nd virial coefficient *B* times molecular weight), both expressed as ml/mg to match the units of concentration. The antibody field has used orthogonal techniques like static light scattering (SLS) to measure a second virial coefficient, BM_1_, and DLS to measure concentration dependence of diffusion, *k*_*D*_. This is typically summarized as *k*_*D*_ = BM_1_ − *k*_*s*_ (Harding and Johnson 1985) suggesting concentration dependence of diffusion involves both a hydrodynamic and thermodynamic component. Many studies combined light scattering, AUC and DLS to investigate the behavior of antibody solutions, emphasizing the complementary information gathered on nonideality and, where present, association (Solovyova et al. 2001; Saluja et al. 2010; Yadav et al. 2011a, b). The goal in part is to survey formulation conditions for the presence of aggregation (Saluja et al. 2010), the avoidance of high viscosity (Yadav et al. 2012), or conditions that favor crystallization (Solovyova et al. 2001). The discussion often focuses on the algebra, do *k*_*s*_ + *k*_*D*_ equal BM_1_, and the problems with subtracting small values, BM_1_ − *k*_*s*_, that nearly cancel. This algebraic expression shown above is valid only in the absence of association. Thus, if *k*_*s*_, *k*_*D*_ and BM_1_ are negative, a nonideal association model should be implemented, such that the nonideality and the association contributions can be accounted for separately. This is generally not feasible by DLS and/or not implemented by SLS methods. The analysis of weak, nonideal association and the extraction of both nonideality and association constants by AUC methods is challenging; it cannot be done with distribution analysis (Rowe 2011; Wright et al. 2018a). On the other hand, rigorous analysis requires nonlinear least squares (NLLS) global fitting of AUC SV data sets to an explicit model that includes both nonideality (*k*_*s*_ and BM_1_) and association. This approach is feasible in SEDANAL because of the ModelEditor feature that allows one to construct any number of simple or complex nonideal associating fitting models (Stafford and Sherwood 2004; Correia and Stafford 2015; Sherwood and Stafford 2016).

Here we present a series of simulations of the impact of *k*_*s*_ and BM_1_ on high concentration antibody solutions of the therapeutic IgG Simponi. We present this over a range of values (0–100 ml/g) individually, *k*_*s*_ and BM_1_ alone, and together, both *k*_*s*_ and BM_1_, to allow a basis set of features that reveal their impact on SV boundary shape. We then choose realistic *k*_*s*_ and BM_1_ values, and simulate a wide range of protein concentrations (1–120 mg/ml) that overlap Simponi’s SC therapeutic dose, 100 mg/ml, and demonstrate the range of data feasible for experimental determinations. Finally, in three recent studies, we showed that all mAb IgG’s weakly self-associate and hetero-associate with other classes of IgG (Wright et al. 2018a, b; Yang et al. 2018). Thus, we present here simulations of nonideal, weak association to demonstrate the challenge of dissecting out basis sets for *k*_*s*_, BM_1_ and association. This analysis has been possible with SEDANAL for a decade. The full power of the method required the implementation of features first described by Todd and Haschemeyer (1981), and this only recently was included in SEDANAL version 6.97 and above. These simulations provide graphical and conceptual insight for AUC users, and encourage the use of rigorous and robust global direct boundary fitting methods for nonideal, weakly associating systems.

## Methods

Simulations were performed with SEDANAL, version 6.97 or later, a software package developed to fit AUC data for complex, nonideal associating systems (Stafford and Sherwood 2004). The models simulated include *k*_*s*_ (hydrodynamic nonideality), BM_1_ (thermodynamic nonideality), and weak self-association, represented here as dimerization, *K*_2_. The mAb modeled is golimumab or Simponi, with a molecular weight of 146,909, an extrapolated *s*^o^ = 6.6 s, an experimentally measured buoyancy (1 − *νρ*) of 0.26623. Simulations in absorbance mode were done with no added noise. Since in silico absorbance or interference experiments are not limited by optical constraints, we adjusted the extinction coefficient for different path cells and present normalized plots. Data were simulated with 1500 points, and output with 650 points between 5.9 and 7.2 cm to match typical absorbance experiments.

Simulations of FDS tracer mode were done by setting the signal strength (extinction coefficient × quantum yield, referred to here as *ε*_1_) for monomer at 1 mg/ml to 1000. To produce a constant signal in tracer mode each apparent extinction coefficient was calculated as 1000/*c*_o_, where *c*_o_ is in mg/ml. For example, cell 2 is 5 mg/ml so *ε*_2_ = 1000/5 = 200; cell 3 is 10 mg/ml so *ε*_3_ = 100; cell 9 is 120 mg/ml so *ε*_9_ = 8.333. All FDS simulations have added Gaussian noise of 10 corresponding to 1% of signal. SEDANAL global fitting of these data sets uses ~ 60–100 scans per data set, which amounts to up to 200,000 total data points, using the Levenberg–Marquardt algorithm to minimize standard deviation. Experimental in vitro or in serum optical constraints are addressed in the discussion.

Values of *k*_*s*_ and BM_1_ were varied over a wide range (0–100 ml/g) to investigate and display their impact on boundary shapes. Experimentally we have shown that Simponi exhibits *k*_*s*_ and BM_1_ values of ~ 10 ml/g (Wright et al. 2018b). (SEDANAL internally uses concentration units of mg/ml and thus *k*_*s*_ and 2BM_1_ have units of ml/mg to make *k*_*s*_*c* or 2BM_1_c dimensionless (Eqs. 1 and 2). However, the field uses ml/g (Rowe 1977; Harding and Johnson 1985; Solovyova et al. 2001; Saluja et al. 2010; Yadav et al. 2011a, b) and thus we plot data vs mg/ml, but report data in units of ml/g (Table 1). This avoids reporting numbers with lots of digits (0.010 ml/mg vs 10.0 ml/g).) Thus, we also separately constrained *k*_*s*_ and BM_1_ to 10 ml/g and varied total concentration from 1 to 120 mg/ml. Weak self- and hetero-association of therapeutic mAbs has been demonstrated (Wright et al. 2018a,b; Yang et al. 2018), and is assumed to be dimerization because of the small extent of reaction. The goal of these simulations was to present graphical basis sets that demonstrate the impact of *k*_*s*_ and BM_1_ on boundary shapes. Thus, simulations are initially presented over a wide range of values to give a picture of what nonideality looks like in an experimental setting. Simulations were also performed at fixed values of *k*_*s*_ and BM_1_ and as a function of concentration (1–120 mg/ml) to mimic therapeutic samples and to establish what is required to extract experimental values of the parameters. We observed that all mAbs self-associate and hetero-associate with other antibodies. To explore this, nonideal association (*k*_*s*_, BM_1_, *K*_2_) simulations as a function of small *K*_2_ values are presented. In addition, all mAbs contain small amounts of dimeric and trimeric aggregates, and these are included in the model to mimic realistic heterogeneity.

**Table 1:** SEDANAL analysis of high concentration FDS data

Concentrations	*s*	*k*_*s*_ ml/g	BM_1_ ml/g	rms
ABC *K*_*s*_ BM_1_ model				
1–120 mg/ml	6.5994 (0.007%)	10.0 (0.025%)	9.99 (0.137%)	14.153
1–40 mg/ml	6.5996 (0.008%)	10.00 (0.06%)	10.00 (0.458%)	14.145
1–20 mg/ml	6.5997 (0.009%)	10.00 (0.10%)	10.04 (0.94%)	14.161

Concentrations	*s*	*k*_*s*_ ml/g	BM_1_ ml/g	*K*_2_ M^−1^	rms
AA_2_BC *k*_*s*_ BM_1_ *K*_2_ model					
1–120 mg/ml	6.5989 (0.013%)	10.00 (0.08%)	10.06 (0.22%)	5008 (0.34%)	14.106
1–40 mg/ml	6.5993 (0.019%)	9.99 (0.17%)	9.93 (0.67%)	4998 (0.38%)	14.116
1–20 mg/ml	6.5991 (0.025%)	10.00 (0.25%)	9.993 (1.94%)	5006 (0.56%)	14.106

ABC model is a monomer-aggregated dimer-aggregated trimer model plus nonideality. AA_2_BC model is the same model plus a reversible monomer–dimer association. Values in parentheses correspond to fractional error determined by a bootstrap analysis and correspond to one standard deviation

All *k*_*s*_ and BM_1_ values are provided as matrices, with elements, *k*_*ij*_ and *B*_*ij*_*M*_*i*_, representing self- and cross-term interactions (Table S1 and S2; Correia and Stafford 2015; Wright et al. 2018a). This represents a family of phenomenological equations for each component that explicitly includes all species concentrations. For example, *s*_1_ (or *s*_*A*_) can be written

3 $${s}_{1}= \frac{{s}_{1}^{\mathrm{o}}}{1+{k}_{11}{c}_{1}+{k}_{12}{c}_{2}+{k}_{13}{c}_{3}}$$

where *c*_2_ corresponds to dimer concentrations, *c*_3_ to trimer concentrations, and *k*_12_ and *k*_13_ are cross-term nonideality terms reflecting the effect of dimer and trimer concentrations on monomer *s*_1_. Other species like reversible dimers or irreversible dimers and trimers have similar expressions (see Supplemental equations S1–S4). In addition, *D*_1_ (or *D*_*A*_) can be written

4 $${\mathrm{D}}_{1} = \frac{{\mathrm{D}}_{1}^{\mathrm{o}} (1+{2B}_{11}{M}_{1}{c}_{1}+{2B}_{12}{M}_{2}{c}_{2}+{2B}_{13}{M}_{3}{c}_{3})}{(1+{\mathrm{k}}_{11}{\mathrm{c}}_{1}+{\mathrm{k}}_{12}{\mathrm{c}}_{2}+{\mathrm{k}}_{13}{\mathrm{c}}_{3} )}$$

with similar expressions for dimers and trimers (Correia and Stafford 2015; Stafford 2016). Note we assume the cross terms *k*_*ij*_ and *B*_*ij*_*M*_*j*_ for dimers and trimers are the same as monomers on a weight scale; this is reasonable since experimentally mAb aggregates have the same frictional ratio, *f*/*f*_o_ (Philo 2003), but this is adjustable in the matrix if required. (See Supplemental Methods section for more details.)

SEDANAL simulates and fits SV data by the method of Claverie (1976) which uses finite-element solutions to the Lamm equation, the partial differential equation that describes simultaneous sedimentation and diffusion in a sector shaped cell (Todd and Haschemeyer 1981).

5 $$(\partial {c}_{i}/\partial t{)}_{r}=-\frac{ \partial }{r\partial r} \left[{c}_{i} {\omega }^{2}{s}_{i}{({c}_{i})r}^{2}-{D}_{i}({c}_{i})r{\left(\frac{\partial {c}_{i}}{\partial r}\right)}_{t}\right]$$

**Fig. 1:**
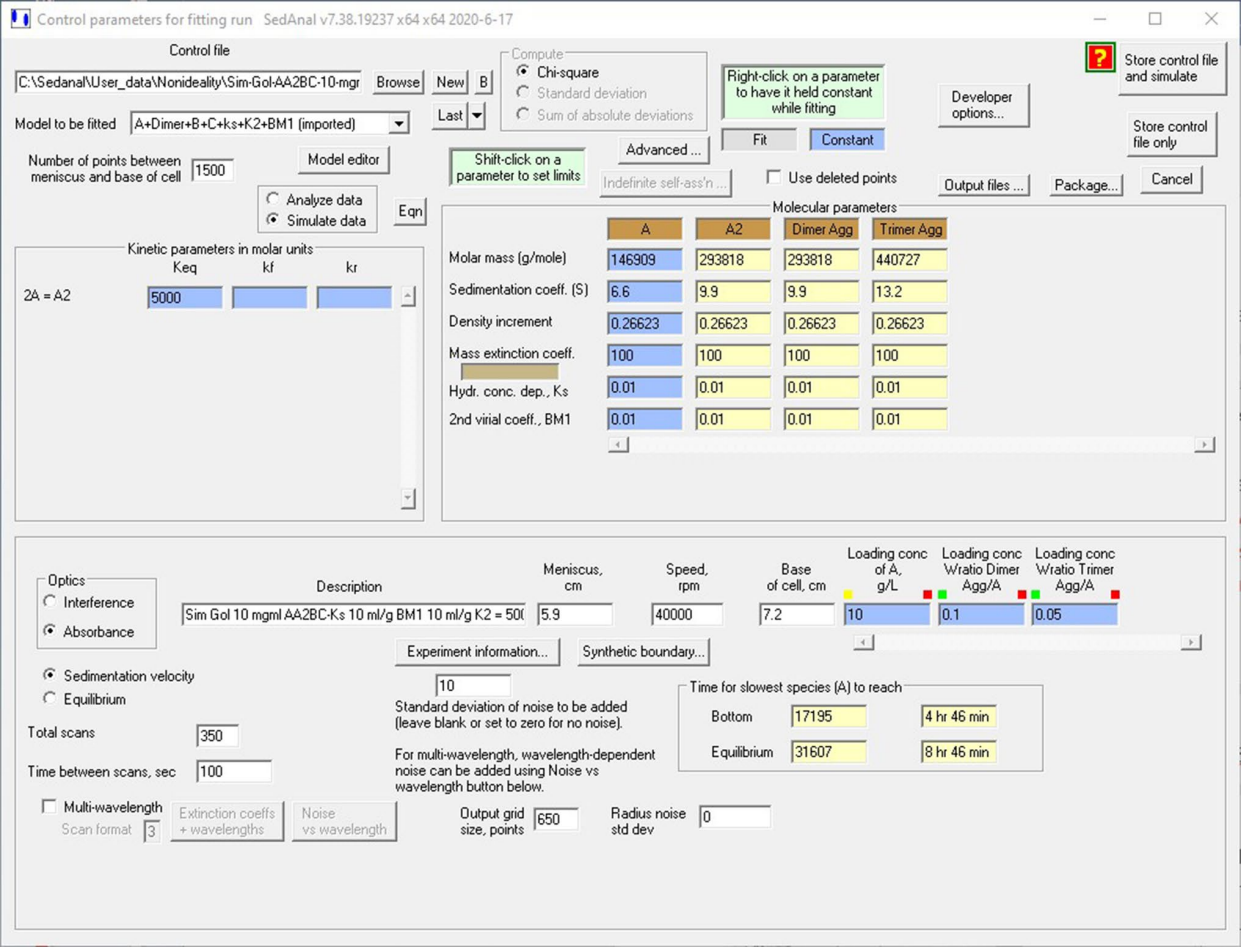
Screen shot of a SEDANAL AUC SV simulation of Simponi at 40 K rpm, 10 mg/ml, with *k*_*s*_ = 0.01 ml/mg, BM_1_ = 0.01 ml/mg, and *K*_2_ = 5000 M^−1^, with 10% dimer and 5% trimer. Other details are visible in the figure

The *s*_*i*_ and *D*_*i*_ terms are concentration-dependent and defined as above (Eqs. 3 and 4) to reflect nonideal behavior. If mAb samples are heterogeneous, paucidisperse systems and contain small amounts of dimeric or trimeric aggregates, simultaneous Lamm equations for the dimer and trimer are solved (Correia and Stafford 2015). Reversible association is included by adding a relationship that links dimer concentration to monomers through equilibrium or kinetic rate constants (Stafford and Sherwood 2004). Figure 1 presents an example of a SEDANAL fitter model for simulation. All antibody samples contain small amounts of irreversible dimer and trimer aggregates, set here to 10% and 5% weight fraction. In addition, antibodies can undergo reversible nonideal association represented here as monomer–dimer (AA_2_) plus *k*_*s*_ and BM_1_. Thus, in this model system, AA_2_ refers to reversible dimer formation and BC or dimer aggregates and trimer aggregates refers to irreversible dimers and trimers.

High concentration solutions to the Lamm equation are intrinsically unstable, primarily due to numerical instability in the base region of the cell (SEDANAL detects this as a “Check Grid” error). Todd and Haschemeyer (1981) were the first to develop curve-fitting methods using Claverie’s rapid numerical solutions to the Lamm equation; the Claverie method was for comparing simulations to real data (Claverie 1976). We constrained parameters to deal with this instability. The most significant of these are defined as *ε* (epsilon) and *η* (eta), where *s*/*s*_o_ = 1 − *ε* and *D*/*D*_o_ = 1 − *η* (Eqs. 3 and 4). For realistic parameters, meaning *k*_*s*_ and BM_1_ equal and positive, *D*/*D*_o_ < 2 (Figure S1A); other choices give less restrictive ranges (Figure S1B). These constraints are provided to the SEDANAL fitter to set the upper limit on *D*/*D*_o_. SEDANAL also allows concentrations at the base to be limited.

We restrict *k*_*s*_ and BM_1_ to positive values because negative values imply association, and therefore, nonideal associating systems are modeled by an explicit nonideal, association scheme (see Fig. 1). Otherwise, the magnitude of *k*_*s*_ and BM_1_ reflect the sum of multiple attractive and repulsive interactions and are difficult to interpret (Laue 2012; Wright et al. 2018b; Laue and Shire 2020). Sedimentation and diffusion coefficients vary across the cell due to nonideality and to local changes in concentration as the boundary components sediment and fractionate (Figure S2). This fractionation or boundary separation has been called demixing by Kingsbury and Laue (2011). This variation in the effect of nonideality is especially true in the base region, where concentrations can increase by two or more orders of magnitude, and calculations of concentration become numerically unstable. SEDANAL has been upgraded to handle these effects (Todd and Haschemeyer 1981), and all the data simulated and fit in this study exhibited stable solutions.

Simulated data are analyzed by Wide Distribution Analysis (WDA) an option in SEDANAL, and plotted as s*g(s*) vs log(s*). This transformation follows from the definition of *g*(s*) = d*c*/d*s*; thus s*d*c*/ds* = d*c*/d(ln(s*)) (Stafford and Braswell 2004; Sherwood and Stafford 2016). WDA was developed for multi-speed experiments but we have found it useful for rapid analysis of single-speed runs as well. We plot data as normalized s*g(s*) vs log(s*) for convenient reading of the scale and for direct comparison of trends in the distributions. The WDA method allows all data scans to be included, with early scans providing information about large components (out to 1000 S in these simulations), and late scans providing information about small species. One can choose different radial positions to view sedimentation at different extents of resolution (Figure S3). A standard DCDT analysis (Stafford 1992) cannot mimic this degree of resolution because it is limited to a narrow span of data scans, in this case a region where the monomer boundary is near 6.4–6.5 cm. Nonetheless, DCDT^+^ analysis (Stafford 1992; Philo 2006) can be compared with s*g(s*) (Figure S4) to reveal the precision and advantages of both methods. DCDT^+^ and WDA generate *s*_w_ and integrated signal area under the curve from results extrapolated to the initial concentration, *c*_o_ (Kegeles and Gutter 1951; Patel et al. 2018). Both methods are used to construct 1/*s*_w_ vs *c* plots (Figs. 6 and 10) to explore the limitations of linear graphical analysis.

## Results

**Fig. 2:**
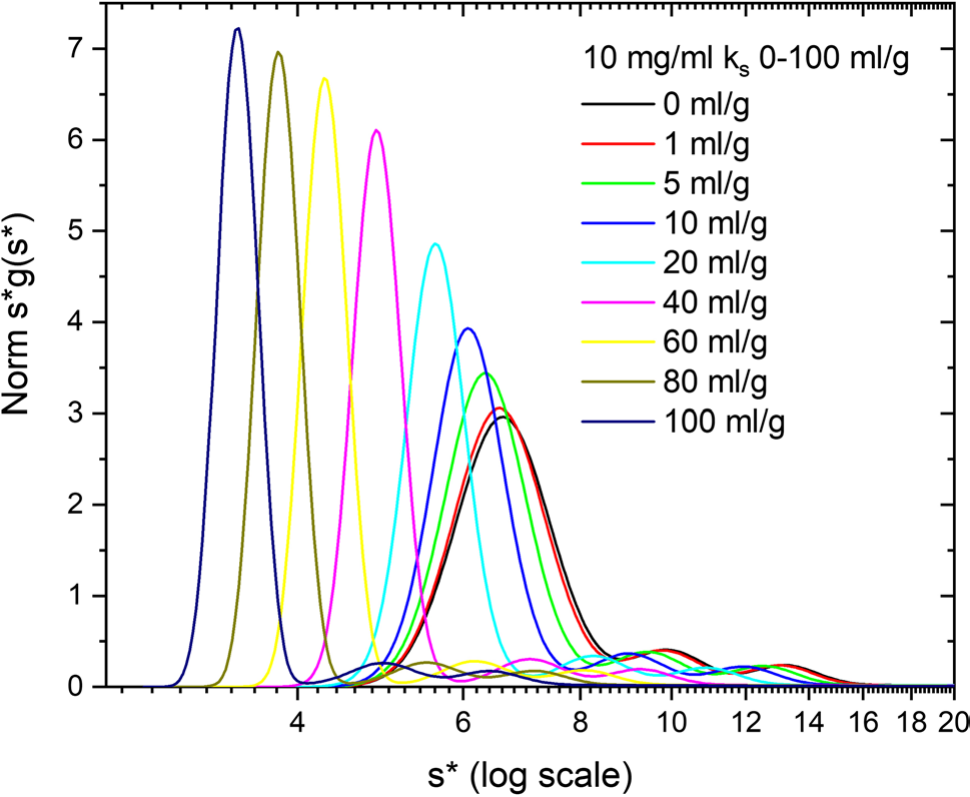
Simulation of ABC or monomer–dimer-trimer model, at 10 mg/ml Simponi plus 10% dimer and 5% trimer irreversible aggregates, where all *k*_*ij*_ values (self and cross term; Table S1) vary from 0 to 100 ml/g. Data are plotted as normalized s*g(s*) vs s* on a log scale

Figure 2 shows a family of simulations performed at 10 mg/ml and over the *k*_*s*_ range 0–100 ml/g. This corresponds to a *k*_11_*c*_1_ range, ml/mg times mg/ml, of 0.01 to 1.0 at *c*_o_, or an initial *s*_app_ range of 6.6–3.3 s. Thus, the monomer boundary slows, and, since (1 + *k*_11_*c*_1_) is also in the denominator for *D* (Eq. 2), the monomer boundary is sharper. Note, the concentrations vary with radial position and time in the simulation due to demixing and radial dilution, and thus the exact *s*_app_ correction is a function of radial position and time throughout the cell and involves contributions from monomer, dimer and trimer species, *s*_*i*_ = (1 + *k*_*i*1_*c*_1_ + *k*_*i*2_*c*_2_ + *k*_*i*3_*c*_3_) (Figure S2). The boundaries for dimer and trimer sediment in the plateau region of the monomer, and are thus dominated by the *k*_*ij*_ cross terms for the larger monomer concentration. Note that the aggregate peak widths do not change significantly because they are in the presence of a nearly constant concentration of monomer. This is in contrast to the monomer boundary where low concentration on the centripetal side of the boundary speeds up sedimentation, and high concentration on the centrifugal side slows sedimentation leading to sharpening, increased concentration and a negative concentration gradient. This is the classic Johnston-Ogston (JO) effect (Johnston and Ogston 1946; Correia et al. 1976, 2016). Oddly enough, even at 10% dimer, the JO effect is evident in the monomer boundary as it interacts with the dimer boundary (Figure S2). The JO effect is more pronounced at both high *k*_*s*_ and high concentrations since it is due to a product, *k*_*s*_*c*.

Typical *k*_*s*_ values for mAbs are 4–11 ml/g (Wright et al. 2018b; Yang et al. 2018). However, experimentally *k*_*s*_ is known to increase dramatically for asymmetric proteins (Creeth and Knight 1965). For example, fibrinogen is 15.5 ml/g; TMV is 33.8 ml/g; myosin is 49–73 ml/g; collagen is 265 ml/g. Pegylated proteins (Li et al. 2012) and IDPs like ELP (Correia et al. 2016) also have very large *k*_*s*_ values. Thus, Fig. 2 demonstrates a wide range of expected behaviors for compact to moderately asymmetric proteins.

**Fig. 3:**
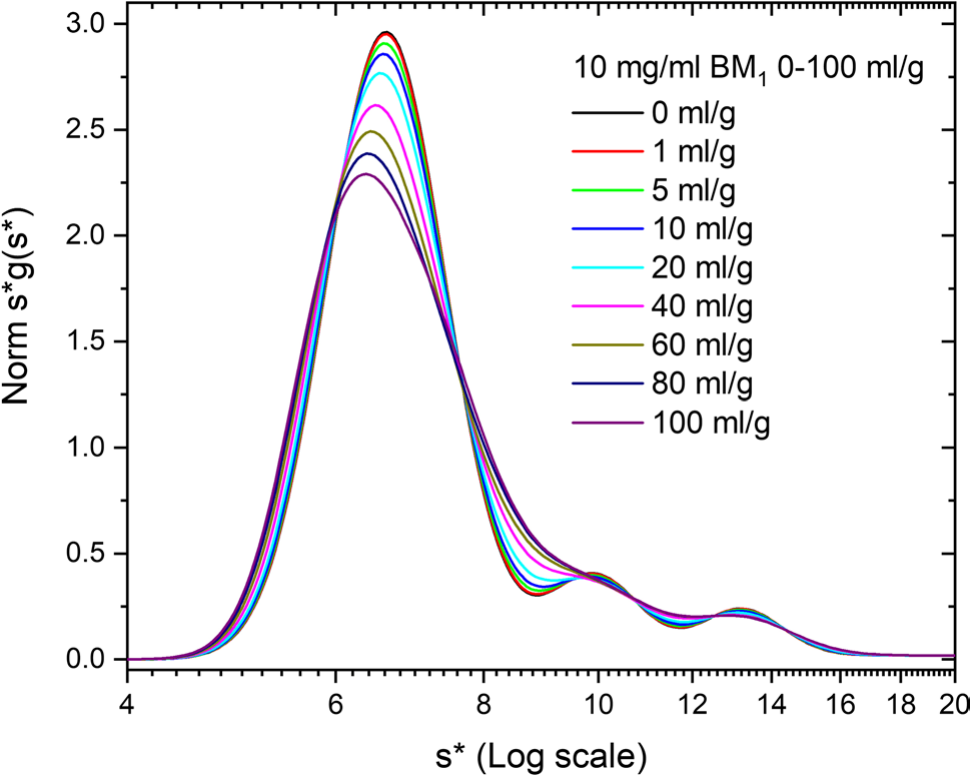
Simulation of ABC model, at 10 mg/ml Simponi plus 10% dimer and 5% trimer aggregates, where all *B*_*ij*_*M*_*j*_ values (self and cross term) vary from 0 to 100 ml/g. Data are plotted as normalized s*g(s*) vs s* on a log scale

Figure 3 shows a family of simulations performed at 10 mg/ml and over the BM_1_ range 0–100 ml/g. Since BM_1_ only influences *D*, the boundary spreads, the peak position drifts, but on average does not sediment differently (*S*_w_ = 7.12 s ± 0.019 s or 0.27%). This corresponds to a 2*B*_11_*M*_1_*c*_1_ range, ml/mg times mg/ml, of 0.02–2.0 at *c*_o_, or an initial *D*/*D*_o_ range of 1–3 corresponding to the (1 + *B*_*ij*_*M*_*j*_*c*_*j*_) term. Note the broadening of the monomer region and an equal degree of broadening of the dimer and trimer regions due to cross term nonideality. This will reduce the resolution of these minor peaks for single sample analysis. Also note this broadening in the monomer region makes the boundary appear more heterogeneous (see “Discussion”).

**Fig. 4:**
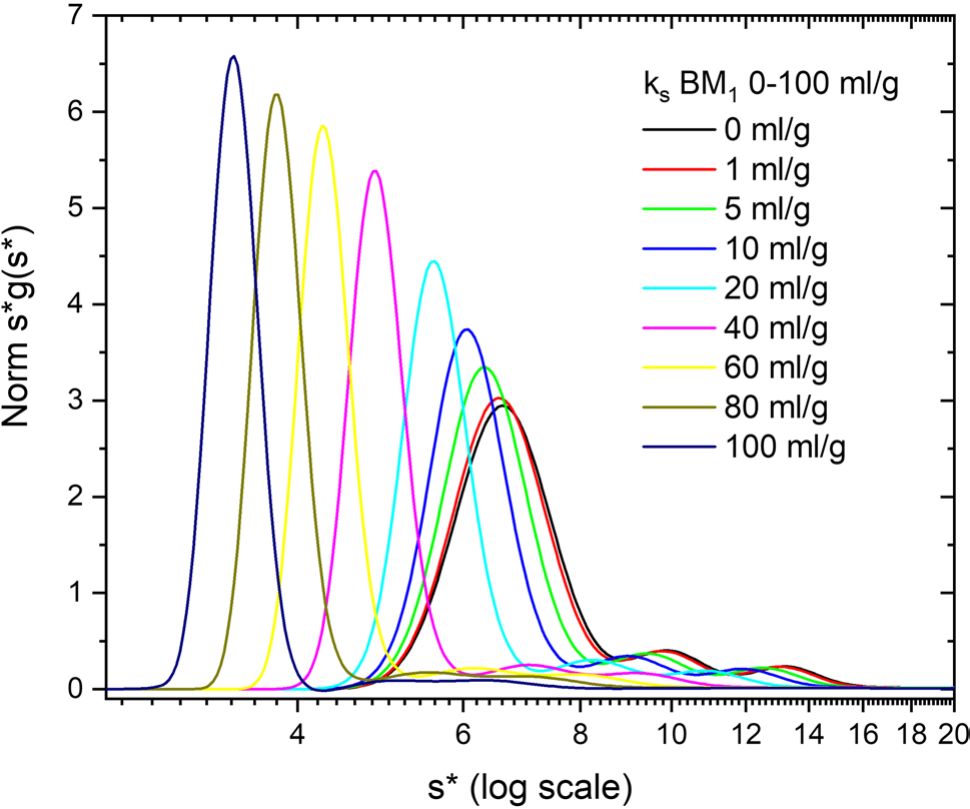
Simulation of ABC model, at 10 mg/ml Simponi plus 10% dimer and 5% trimer aggregates, where all *k*_*ij*_ and *B*_*ij*_*M*_*j*_ values (self and cross term) vary from 0 to 100 ml/g. Data are plotted as normalized s*g(s*) vs s* on a log scale

Both *k*_*s*_ and BM_1_ are caused by a combination of excluded volume, charge and shape effects (Tanford 1961; Rowe 1977). As discussed above and in the literature, *k*_*s*_ and BM_1_ have similar magnitudes, and thus this range of values is reasonable in principle (Yadev et al. 2012; Wright et al. 2018b; Chaturvedi et al. 2019; Chaturvedi and Schuck 2019). To demonstrate this similarity we simulate 10 mg/ml Simponi and vary both *k*_*ij*_ and *B*_*ij*_*M*_*j*_ equally from 0 to 100 ml/g (Fig. 4). This range of parameter values should impact *s* values in a manner similar to Fig. 2, but *D* values now have changes to both the numerator and denominator that offset each other (Eq. 4). In this case the ratio (1 + *B*_11_*Mc*_1_)/(1 + *k*_11_*c*_1_) will vary from 1 to 1.5, but as described above the concentrations of all species vary with radial position and time in the run due to demixing and radial dilution, and thus the exact *D*/*D*_o_ value is a function of radius and time throughout the cell (Eq. 4). This increase in *D*/*D*_o_ causes the dimer and trimer regions to broaden and thus reduces the resolution of the dimer and trimer peaks. Note also that these data exhibit a JO effect at the monomer–dimer interface in a concentration-dependent manner according to the value of *k*_*ij*_ cross terms (Figure S2).

**Fig. 5:**
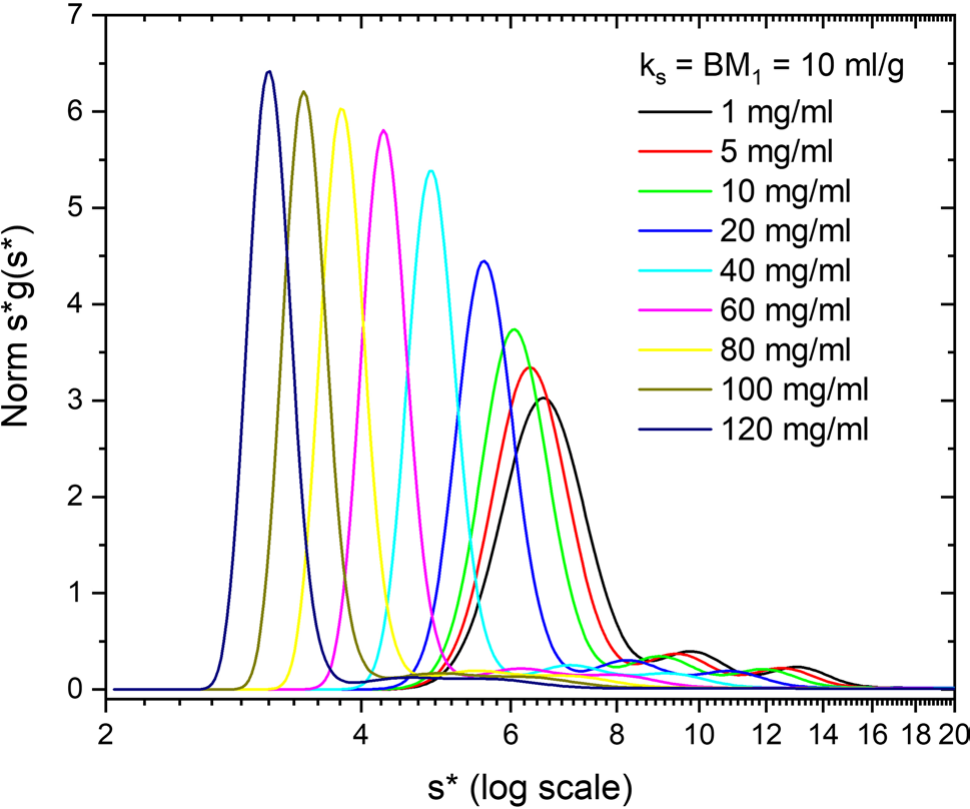
Simulation of ABC model, at 1 to 120 mg/ml Simponi concentrations plus 10% dimer and 5% trimer aggregates. Nonideality parameters *k*_*ij*_ and *B*_*ij*_*M*_*j*_ values (self and cross term) are constrained to 10 ml/g. Data are plotted as normalized s*g(s*) vs s* on a log scale

These first three sets of simulations (Figs. 2, 3 and 4) vary nonideality at a fixed protein concentration. Figure 5 shows concentration-dependent simulations of Simponi from 1 to 120 mg/ml plus 10% dimer and 5% trimer aggregates. (Figure S6A presents the DCDT^+^ *g*(s*) vs s* version of this plot). Nonideality parameters, *k*_*ij*_ and *B*_*ij*_*M*_*j*_, (self and cross term) are constrained to experimentally reasonable values of 10 ml/g (Wright et al. 2018a). The increase in hydrodynamic nonideality reduces *s*_app_ in a manner similar to Fig. 2. The ratio of thermodynamic to hydrodynamic terms (1 + 2*B*_*ij*_*M*_*j*_*c*)/(1 + *k*_*ij*_*c*) in the plateau region of the simulations increases *D*/*D*_o_ from 1.01 to 1.58. This is most evident in the dimer and trimer zones where discrete peaks are broadened and more difficult to resolve. As the protein sediments the concentration dramatically increases at the base (Figure S5A), causing significant spreading or back diffusion in the base region of nonideal samples that precludes using radial values too close to the base for WDA (Figure S5B). (It is worth noting that filling a cell to only 6.1 or 6.2 cm reduces the usable radial range for analysis and may be problematic for these highly nonideal systems, especially in FDS experiments where the base is already cutoff optically.)

These simulations emphasize three features of highly, nonideal systems due to either large *k*_*ij*_ values or high concentrations. First, the boundary will be hyper-sharp during sedimentation, due to concentration dependence of *s*_app_, and second, the base region will be very broad due to back diffusion into the plateau region caused by an increase in *D*_app_. This second feature is entirely due to thermodynamic nonideality and reduces the radial region of data that can be analyzed by typical software packages. Thirdly, minor aggregates typically found in mAb solutions will experience significant broadening making their determination challenging for single sample analysis. In more heterogeneous solutions like serum, the high concentrations at the base cause density gradients that lead to banding of some lipoprotein species. (Analysis of serum samples by AUC FDS methods will be discussed in a future paper). It is worth noting that Figs. 4 and 5 look identical in normalized mode, but Fig. 5 is a concentration series, and thus the signal actually increases dramatically unless done in tracer mode (see below).

**Fig. 6:**
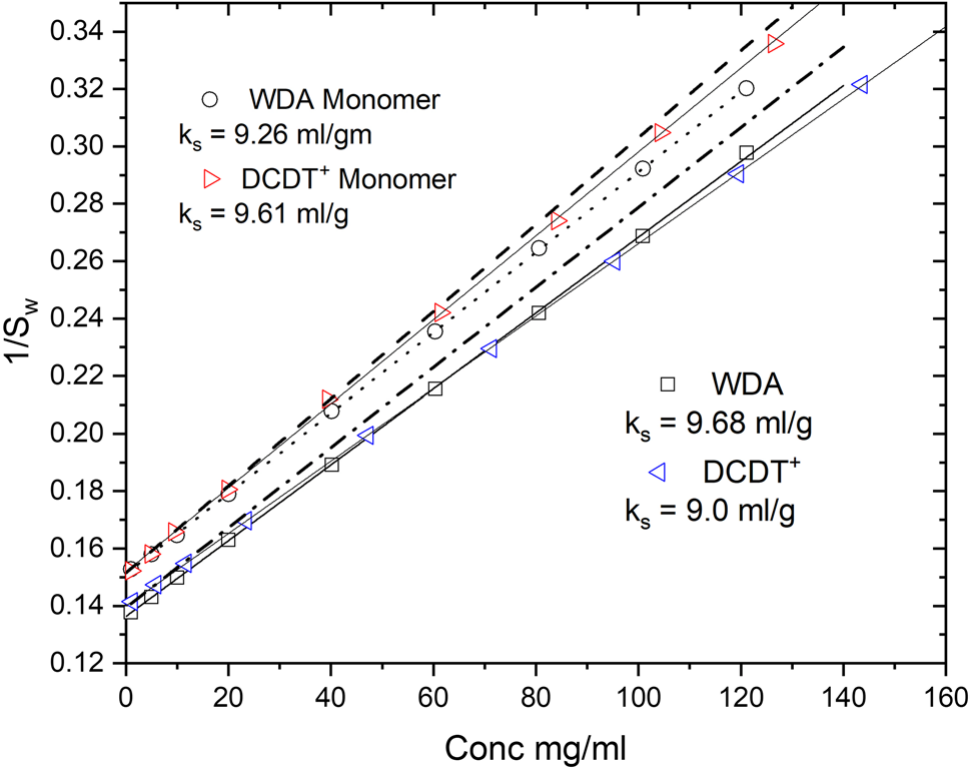
1/*s* vs *c* plots of the data in Fig. 5 analyzed with DCDT^+^ or WDA. The heavy dashed line represents monomer with an intercept at 6.6 s and *k*_*s*_ = 10 ml/g. The heavy dot-dash line corresponds to a mixture of monomer–dimer-trimer with a *s*^o^_w_ = 7.174 and *k*_*s*_ = 10 ml/g

AUC data collected as a function of concentration are typically analyzed by linear plots of *s* vs *c* or 1/*s* vs *c* to extract *k*_*s*_. The first plot is based upon the equation *s* = *s*^o^(1 − *k*′_*s*_*c*), a Taylor expansion of Eq. (1), while the second plot is the linear rearrangement of Eq. (1); 1/*s* = 1/*s*^o^ + (*k*_*s*_/*s*^o^)*c. We have previously discussed the relationship between these two coefficients, *k*_*s*_ and *k*′_*s*_ (Wright et al. 2018b). A 1/*s*_w_ vs *c* plot of the data in Fig. 5 is presented in Fig. 6 using *s*_w_ of the full boundary, the WDA monomer peak positions or DCDT^+^ *s*_w_ of the monomer region. The data from WDA and DCDT^+^ are plotted vs total concentrations or concentrations corrected for radial dilutions (Patel et al. 2018). Matching the *s*_w_ with the appropriate concentration is the challenge in the analysis of mixtures by a graphical method. The extrapolated *s*^o^ for monomers are reasonably close to the simulated value 6.6 S. The *k*_*s*_ values approach the expected value of 10 ml/g but cannot precisely capture the more dynamic nonideality effect of radial dilution and demixing of the species (see “Discussion”). A simulation of just monomers (0% dimers, trimers) gives better but also not perfect estimates of *k*_*s*_ (data not shown). The *s*_w_ data for the full boundary also approach the correct *s*^0^_w_ and *k*_*s*_ values, but fail to capture the dynamics of nonideality and demixing (Kegeles and Gutter 1951; Patel et al. 2018).

**Fig. 7:**
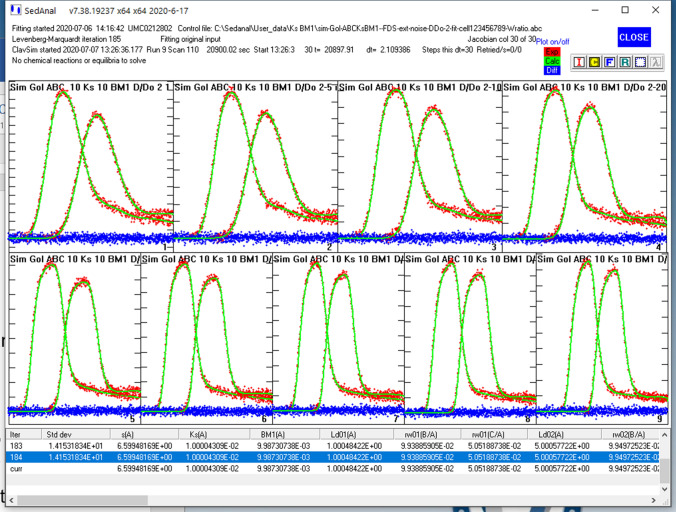
A global SEDANAL fit of nine FDS data sets to an ABC-*k*_*s*_-BM_1_ model with 10% dimer and 5% trimer aggregates. Data were simulated with a tracer amount of labeled Simponi corresponding to 1000 counts of monomer, and increasing amounts of unlabeled mAb. To produce a constant signal in tracer mode each apparent extinction coefficient is set to 1000/*c*_o_ where *c*_o_ is in mg/ml. In this in silico FDS tracer mode, extinction coefficient is held constant and concentration is fit

Choosing the appropriate concentration and *s*_w_ seems to be the challenge in this linear analysis (Patel et al. 2018). To rigorously deal with sedimentation, radial dilution and species demixing (Kingsbury and Laue 2011), the data should be globally fit to an appropriate model as a function of concentration and time using finite-element solutions to the Lamm equation. To demonstrate the need for global fitting, we simulated FDS tracer data for this same *k*_*s*_, BM_1_ model over the concentration range of 1–120 mg/ml. A global SEDANAL fit of the data is presented in Fig. 7. The best fit returns *s* = 6.5994, *k*_*s*_ = 10.0 ml/g and BM_1_ = 9.99 ml/g with an rms = 14.153 which is the expected value for the added noise, 10, increased by the √2 for the ΔC method used by SEDANAL. All the dimer and trimer weight ratios are correct within 1%. Our conclusion is that a full concentration series, analyzed by global direct boundary fitting with a proper *k*_*s*_, BM_1_ heterogeneous model is the most rigorous and accurate way to analyze high concentration AUC SV data. A bootstrap analysis of these data reveals very tight error bars as expected for simulated data (with 217,450 points; Table 1), but the error bars for BM_1_ are ~ 6 × larger than for *k*_*s*_. This is consistent with our experimental observation that BM_1_ is much more difficult to measure than *k*_*s*_ by SV analysis.

**Fig. 8:**
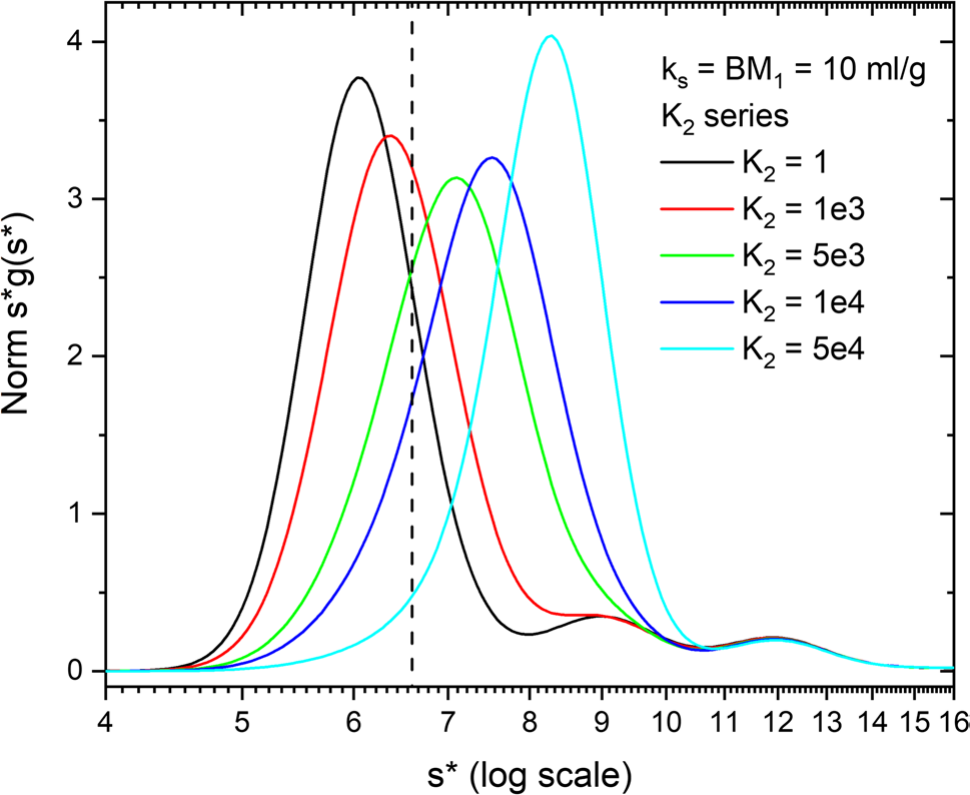
Simulation of nonideal self-association models as a function of *K*_2_ at 10 mg/ml, *k*_*s*_ = BM_1_ = 10 ml/g. Results for *K*_2_ values of 0, 1000, 5000, 1e4, and 5e4 M^−1^ are presented. (Note these *K*_2_ values in M^−1^ correspond to *K*_*d*_ of 0, 73.4, 14.7, 7.3 and 1.47 mg/ml, or *K*_2_ (mg/ml)^−1^ = 2 × *K*_2_ (M)^−1^/146,909) The monomer *s* value is indicated by the vertical dashed line

We observed that all mAbs exhibit weak self-association properties (Wright et al. 2018a, b; Yang et al. 2018). To include this in the analysis we have simulated a nonideal self-associating model at 10 mg/ml and increasing values of *K*_2_ (Fig. 8). At 10 mg/ml and no association (*K*_2_ = 0) the curve clearly exhibits nonideality relative to the known monomer s value. At 1000 M^−1^ the main peak is still slightly nonideal meaning the monomer peak runs at less than 6.6 s. The impact of this is that *k*_*s*,app_ will be smaller than expected because it is masked by the association (Wright et al. 2018a; Yang et al. 2018). As *K*_2_ increases the main peak shifts to the right indicating association, although it is worth noting the boundary is still nonideal, but now the nonideality is partially masked by weak association.

**Fig. 9:**
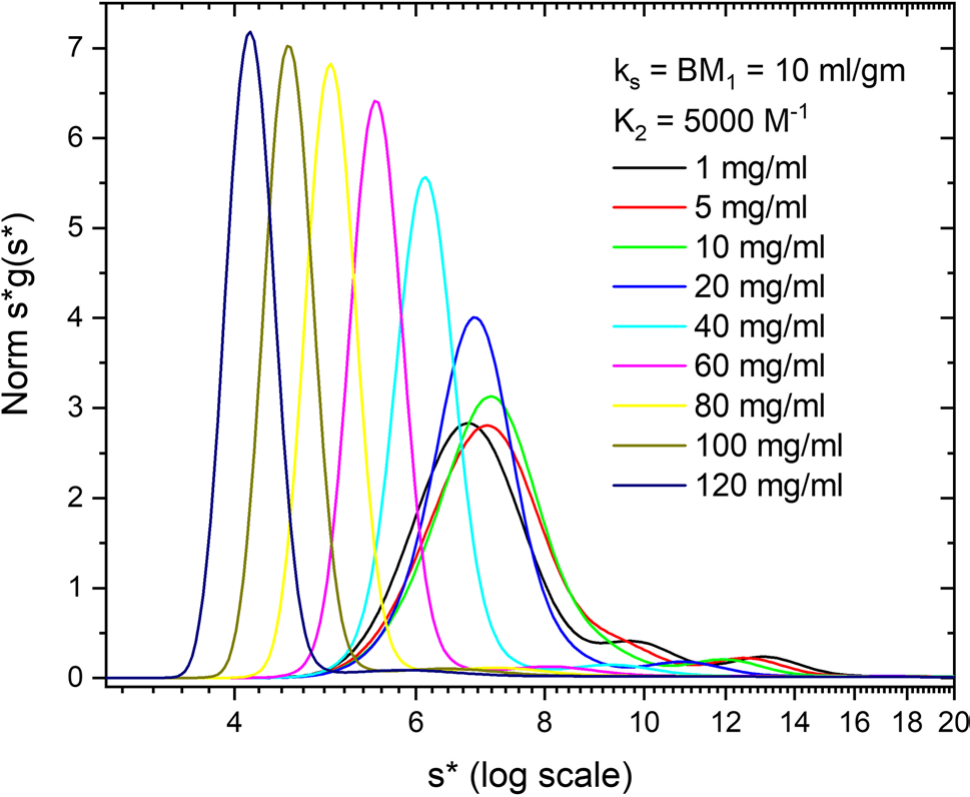
Simulation of nonideal self-association (*k*_*s*_ = BM_1_ = 10 ml/g, *K*_2_ = 5000 M^−1^) as a function of concentration (1–120 mg/ml). The data are plotted as Norm s*g(s*) vs s* on a log scale

**Fig. 10:**
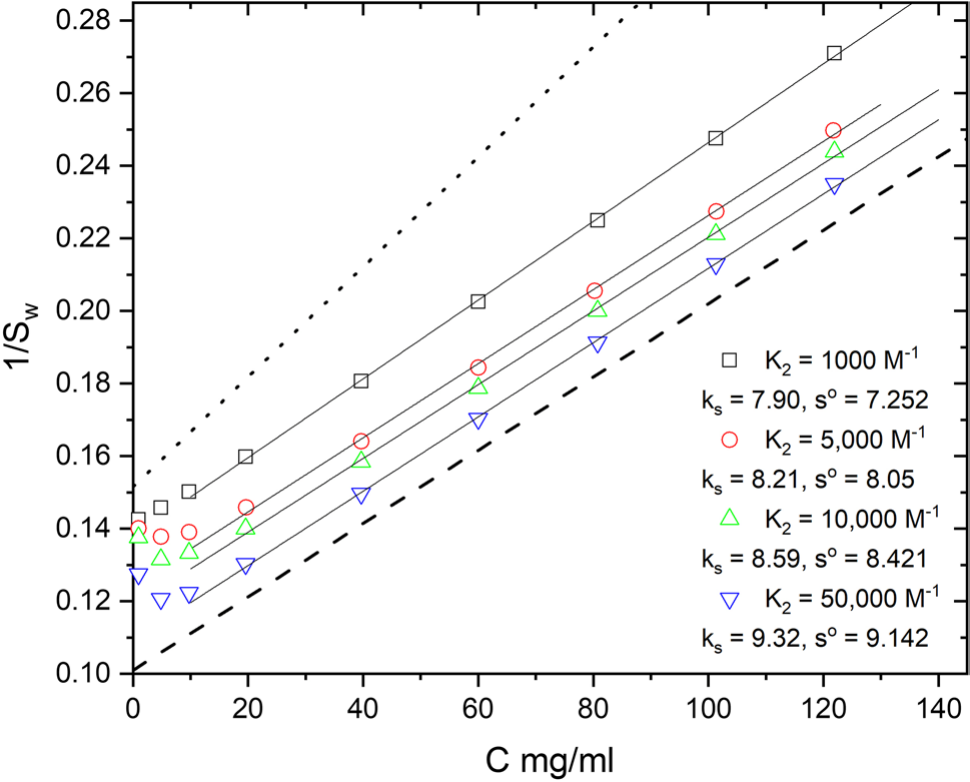
A plot of 1/*s*_w_ vs concentration for *k*_*s*_, BM_1_, *K*_2_ data in Fig. 9 and repeated for data simulated with *K*_2_ = 1000, 1e^4^ and 5e^4^ M^−1^. The data were analyzed by integration of the full boundaries with DCDT^+^. The linear portions of the curves were fit to a straight line to extract apparent *k*_*s*_ values and extrapolated *s*^o^ values. The dotted line is pure monomer with *s*^o^ = 6.6 S and *k*_*s*_ = 10 ml/g. The dashed line is pure dimer with *s*^o^ = 9.9 S and *k*_*s*_ = 10 ml/g

To investigate this further we simulated a nonideal concentration series for all four *K*_2_ values (Fig. 9 presents data for *K*_2_ = 5000 M^−1^) and then plotted all the data as 1/*s*_w_ vs *c*, corrected for radial dilution (Fig. 10). Up to ~ 10 mg/ml the data shifts to increasing *s* values, indicating association, and then shifts to lower *s* values reflecting an increasing effect of nonideality (Stafford 1980). For weak association, the linear portions of the curve give a reduced apparent *k*_*s*_ value consistent with masking of nonideality by association (Wright et al. 2018a). Above 20 mg/ml nonideality becomes dominant, and with increasing *K*_2_ values, the slope and intercept approach but do not equal *k*_*s*_ and *s*^o^ values expected for the dimer. Nonideality and association are concentration-dependent and thus give different apparent answers at different concentrations. These results support the overlapping impact of radial dilution and fractionation during sedimentation on nonideality and association and the challenge of extracting these values by linear graphical methods. As stressed above, the rigorous analysis of these data requires NLLS global fitting to the primary SV data.

**Fig. 11:**
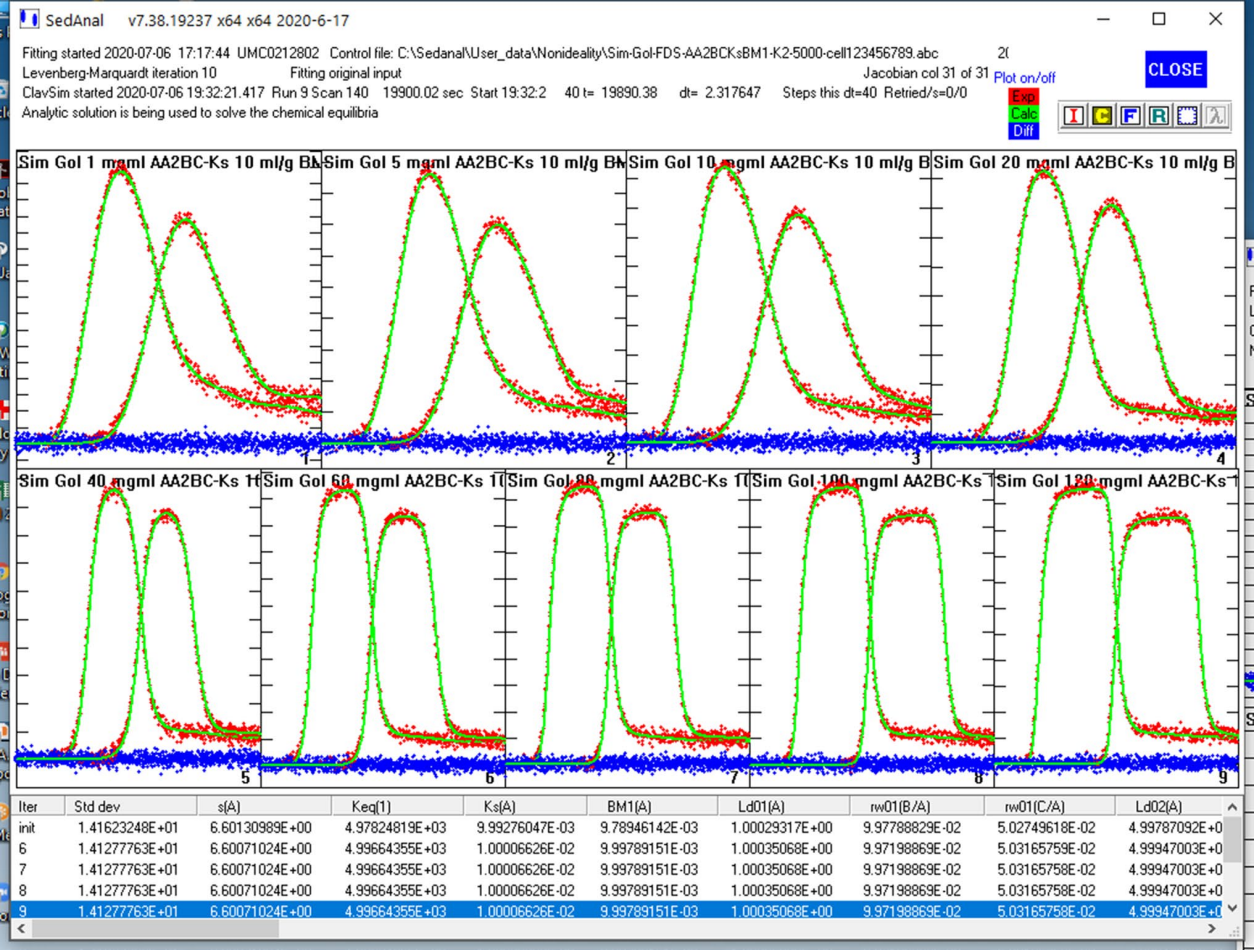
A global SEDANAL fit of nine FDS data sets to an AA_2_BC-*k*_*s*_-BM_1_-*K*_2_ model with 10% dimer and 5% trimer aggregates and *K*_2_ = 5000 M^−1^. Data were simulated with a tracer amount of labeled Simponi corresponding to 1000 counts of monomer, and increasing amounts of unlabeled mAb. To produce a constant signal in tracer mode each apparent extinction coefficient is set to 1000/*c*_o_ where *c*_o_ is in mg/ml

To demonstrate the requirement for NLLS fitting, we simulated FDS tracer data for this same *k*_*s*_, BM_1_, *K*_2_ model over the concentration range of 1–120 mg/ml for a nonideal system with *K*_2_ = 5000 M^−1^ (Fig. 11). The best global fit of these data returns *s* = 6.5989, *k*_*s*_ = 10.00 ml/g and BM_1_ = 10.06 ml/g, *K*_2_ = 5008 M^−1^ and an rms = 14.106. The weight ratio of dimer and trimer aggregates are returned correctly to less than 1%. Bootstrap analysis reveals a 3 × larger uncertainty on BM_1_ vs *k*_*s*_ consistent with the observation above (Table 1; 184,340 points). The BM_1_ and *K*_2_ data have a surprisingly small correlation coefficient (*R* = 0.34). Thus, we conclude that rigorous analysis of high concentration, nonideal, weak, associating systems requires global direct boundary fitting. At present only SEDANAL does global direct boundary analysis of AUC SV data to a nonideal associating model over this concentration range. There are other AUC approaches to analysis of high concentration SV data (Chaturvedi et al. 2018, 2019; Chaturvedi and Schuck 2019) that use a nonideal version of the *c*(*s*) method *c*_NI_(*s*). These methods estimate *k*_*s*_ by a plot of *s*_w_ versus concentration, a problematic approach as discussed above, and do not do global fitting. Combining sedimentation equilibrium analysis to independently measure BM_1_ is certainly a reasonable approach, but it is not as useful for heterogeneous solutions like serum (see “Discussion”). Examples of additional fitting requirements for real experimental data will be presented in a separate publication.

## Discussion

The goal of this work is to present graphical distributions from SV simulations of nonideal mAbs solutions at high concentrations up to and exceeding the therapeutic concentration. This allows extraction of features that are direct consequence of hydrodynamic and thermodynamic nonideality. This should provide the opportunity to see the impact of *k*_*s*_ and BM_1_ in SV data sets. Is the boundary hypersharp and running more slowly than expected? Is the base region much broader than typically observed? Is there evidence of negative gradients in the plateau region because of the JO effect, consistent with the presence of mixtures? The phenomenological or empirical parameters *k*_*s*_ and BM_1_ are best determined from their concentration-dependence. It is imperative that experiments be done as a function of loading concentration, the wider the range the better. We simulated data up to 120 mg/ml to exceed the therapeutic dose of Simponi. We also explored the range of 1–100 ml/g for *k*_*s*_ and BM_1_. As discussed above, this range corresponds to compact, globular and moderately asymmetric proteins.

mAbs appear to have *k*_*s*_ values in the range of 4–11 ml/g (Wright et al. 2018a, b; Yang et al. 2018), but this is sensitive to conditions, especially salt concentration, and methods of analysis. Weak self-association will mask *k*_*s*_ and BM_1_ values, and thus proper models that include both nonideality and association are required (Wright et al. 2018a). Strongly associating mAbs are rare, but there are reports of much larger *k*_*s*_ and BM_1_ values that may reflect asymmetric complex formation, linear chains of mAbs in an extended conformation (Liu et al. 1995; Hopkins et al. 2018). This discussion is based upon the assumptions outlined by Rowe (1977) that *k*_*s*_ is due to swollen volume *V*_*s*_ and *f*/*f*_o_ effects according to the equation *k*_*s*_ = 2*v*(*V*_*s*_/*v* + (*f*/*f*_o_)^3^) where *v* is vbar. The effects of pH and salt on *k*_*s*_ are also of great interest. Yang et al. (2018) showed that increasing salt suppresses mAb association and allows *k*_*s*_ values to increase and approach values of 10 ml/g. Recently Chaturvedi et al. (2019) and Connolly et al. (2012) reported *k*_*s*_ values > 20 ml/g in low salt conditions. Many investigations vary salt concentration and observe a suppression of association and reduction in viscosity (Yadev et al. 2012). Changes in pH have a surprisingly small impact. Direct charge measurements by membrane-confined electrophoresis (Moody and Shepard 2004) show a rather constant mAb charge as a function of pH. They speculate that mAbs buffer charge due to Cl^−^ or anion binding (Yadav et al. 2012; Yang et al. 2018).

The simulations presented here were done in absorbance mode in the absence of added noise. Interference could have worked equally well because in silico has no optical limitations. In practice absorbance experiments on typical mAbs at 280 nm, assuming a generous useable range of 2 OD, are limited to ~ 1.1 mg/ml in a 12 mm cell, to ~ 13 mg/ml in a 1 mm cell. Collecting data at the UV minimum near 254 nm will expand this range, depending upon Trp/Tyr content, by a factor of 2. Interference has a much wider dynamic range and can be useful for AUC SV experiments up to 50 mg/ml (Chaturvedi et al. 2018). Nanolytics has produced a new interference system (Schilling and Krause 2015) that claims to work up to 150 mg/ml. Both of these claims about interference optics are dependent upon accurate optical focusing and conditions, especially speed, since fringe resolution is strongly dependent upon the gradient steepness. As shown in Figure S3, resolution of sedimenting species is strongly speed-dependent. Conditions also imply proper dialysis, the use of meniscus matching centerpieces, and may be influenced by formulation buffers. It is worth pointing out that both absorbance and interference are mostly limited to single component systems like pure mAbs.

Our approach to high concentration experiments uses FDS in a tracer mode, referred to as BOLTS or Biological On-Line Tracer Sedimentation by Laue (2009), where an Alexa-488 labeled mAb is run in a high concentration background of unlabeled mAb (Wright et al. 2018a). This allows no significant limit to the concentration ranges explored, besides solubility, and has the further advantage of being useful in serum, cell, and tissue extracts. As described above, this approach also allows a constant fluorescence signal to be used and thus provides better signal/noise characteristics for data analysis (Husain et al. 2015; Lyons et al. 2013). Our preference is ~ 500–1000 counts (out of 4096 max), which is a function of label efficiency, but generally means 100–200 nM in typical mAb samples. This is the range of FDS data we simulate and fit in Figs. 7 and 11. The results are remarkably good with excellent accuracy and precision. Not all mAb are available to typical users over a range of 1–120 mg/ml. Thus experiments may only be possible over 1–40 or 1–20 mg/ml ranges. Fitting of these more limited concentration ranges also gives excellent results with the expected slightly wider uncertainty limits, especially in BM_1_ (Table 1). These considerations will be explored in subsequent experimental work.

It has been standard practice in the AUC field to extract *k*_*s*_ values from concentration dependent SV analysis mostly through linear plots (Figs. 6 and 10; Kegeles and Gutter 1951; Creeth and Knight 1965; Rowe 1977; Li et al. 2012; Patel et al. 2018; Wright et al. 2018a, b). Although it has been proposed (Solovyova et al. 2001; Wright et al. 2018a, b), it is less common to extract BM_1_ values from SV analysis. Thus, it is worth asking, what is the consequence of not including BM_1_ in a direct boundary fit of data like Figs. 7 and 11? To investigate this we refit the AA_2_BC data in Table 1 without BM_1_. The 1–120 mg/ml data set returned an increase in rms (rms/rms_o_ where rms_o_ is the fit with BM_1_) of 23%, while for the 1–40 mg/ml data the rms/rms_o_ increased by 3.5%, and for the 1–20 mg/ml data the rms increased by only 0.8%. To achieve these fits the *s*^o^ values increased slightly while *K*_2_ decreased (not shown). The surprising result was that the aggregated dimer to monomer fraction, *B*/*A* in the model (Fig. 1), increased dramatically while the monomer fraction decreased. As mention above for Fig. 3, the impact of BM_1_ is to broaden the boundary making it appear heterogeneous. Thus, in the absence of BM_1_ the best fit now increases *B*/*A*, the aggregated dimer/monomer fraction, to match the apparent heterogeneity of the boundary shape. Thus, not including BM_1_ in the fit appears to make aggregation seem to increase. To investigate the coupling between BM_1_ and the other parameters, we repeated the simulations without aggregated dimers and trimers, i.e. without species B or species C, and then compared fitting with and without BM_1_ (Table 2). Now the rms/rms_o_ values were increased by 5.5%, 19% and 90%, respectively, for the three concentration ranges. This was also matched by slightly larger s^o^ values and smaller *K*_2_ values, with *s*^o^ and *K*_2_ values highly correlated, *R* = 0.85. Thus, in the absence of aggregated dimers, the omission of BM_1_ in the fit has a larger impact on the best NLLS fit. These large rms/rms_o_ deviations are consistent with weak correlation between BM_1_ and the other parameters in the full fits (*K*_2_ vs BM_1_, *R* = 0.12; *s* vs BM_1_, *R* = 0.18; *k*_*s*_ vs BM_1_, *R* = 0.14). Since BM1 seems to be nearly orthogonal to the other parameters, *s*, *k*_*s*_ or *K*_2_ cannot compensate for the absence of BM_1_ in the fit. In practice, this will be complicated by the actual presence of molecular heterogeneity due, for example, to variable glycosylation states or mixtures of other mAb conformations.

**Table 2:** SEDANAL analysis of AA_2_ *k*_*s*_ BM_1_ *K*_2_ = 5000 FDS model

Concentrations (mg/ml)	*s*	*k*_*s*_ ml/g	BM_1_ ml/g	*K*_2_ M^−1^	rms
1–120	6.6007 (0.014%)	9.9995 (0.02%)	9.9805 (.17%)	4989.3 (0.22%)	14.13
	6.7089 (0.023%)	10.26 (0.05%)	–	3723 (0.45%)	26.884 (1.902)
1–40	6.6008 (0.015%)	10.001 (0.045%)	9.9419(.529%)	4989.3 (0.272%)	14.128
	6.6333 (0.015%)	9.9618 (0.099%)	–	4407.3 (0.334%)	16.781 (1.188)
1–20	6.6005 (0.015%)	10.011 (0.14%)	9.9126(1.36%)	44998.8 (0.368%)	14.131
	6.6210 (0.016%)	9.6143 (0.159%)	–	4444.2 (0.395%)	14.905 (1.055)

AA2 model is a reversible monomer–dimer association with nonideality *k*_*s*_ and BM_1_. To explore the cross-correlation of BM_1_ with *k*_*s*_ and *K*_2_, we re-simulated without species B or C, meaning without aggregated dimer-aggregated trimer. Values in parentheses correspond to fractional error determined by a bootstrap analysis and correspond to one standard deviation. Note the ratio of best fit rms to the rms_o_ of the fit with BM1 (values in parentheses under the rms column) is significantly larger without aggregated B and C included in the model to compensate for heterogeneity (see “Discussion”). These large rms/rms_o_ deviations are consistent with weak correlation between BM_1_ and the other parameters in the full fits (*K*_2_ vs BM_1_, *R* = 0.12; *s* vs BM_1_, *R* = 0.18; *k*_*s*_ vs BM_1_, *R* = 0.14). Since BM1 seems to be orthogonal to the other parameters, *s*, *k*_*s*_ or *K*_2_ cannot compensate for the absence of BM_1_ in the fit

As previously reported, the FDS dynamic range in our hands is low nM to low µM. Linearity of signal is the main concern (Lyons et al. 2013). In the case of associating systems, constant ratio of signal/mg upon association is assumed but must be proven. The main concern with using labeled samples is that the label does not interfere with the molecular behavior. It is possible that label–label interactions occur in complexes that quench or enhance the signal intensity. This is not usually seen in tracer experiments; experiments with 100% labeled material are more likely to exhibit quenching behavior depending upon the locations or separation of the labels in oligomeric complexes. (An example of quenching in FDS data will be presented elsewhere.)

The nonideality equations used here (1–4) are referred to as phenomenological equations meaning empirical or experimental. Thermodynamic nonideality, BM_1_, refers to excluded volume (Tanford 1961) and charge effects as described by the Donnan term in osmotic pressure (Scatchard 1946) or sedimentation equilibrium experiments (Roark and Yphantis 1971). Hydrodynamic nonideality, *k*_*s*_, also refers to excluded volume and charge effects as reflected in the backflow during an SV experiment where displaced solvent must replace the volume a macromolecule vacates during sedimentation. The sedimenting particle has an effective solvated volume *V*_*s*_ that reflects hydration, shape through an *f*/*f*_o_ term, and the entrained solvent captured within the Debye length (Fuoss 1959; Fuoss and Onsager 1932; Rowe 1977, 1992). It is worth noting that the effective charge is much smaller than typically assumed due to charge screening and anion binding (Laue 2011; Laue and Shire 2020). This in principle reflects the Stokes radius of the effective electroneutral sphere. We are aware that a good *k*_*s*_ model system that controls for shape and charge would be useful here for investigating the dependence of *k*_*s*_ on charge. Our purpose is not to assign theoretical values but rather to present the impact of typical empirical values for these parameters. It is more important that the impact of these values is graphically presented, and that their measurement by NLLS fitting to proper models, nonideality plus association, be clearly outlined.

It is generally assumed that the correct concentration scale for high concentration work is volume fraction (Ross and Minton 1977) and recent AUC studies on high concentration use the volume fraction *Φ* scale (Chaturvedi et al. 2018; Chaturvedi and Schuck 2019). The conversion is in principle a linear transformation, as simple as *νc* (Broide et al. 1991), but more realistically *V*_*s*_*c* where the swollen or effective volume is used (Rowe 1992; Chaturvedi and Schuck 2019). At high volume fraction the shape and packing considerations may become important for mAbs (Garidel et al. 2017). The conversion of weight concentration to volume fraction can be verified from intrinsic viscosity measurements: ${\Phi }_{\mathrm{eff}}=[\eta ]c/2.5$, where 2.5 is for spheres (Cantor and Schimmel 1980). Therefore, we are currently making viscosity measurements on mAbs and serum proteins and will present those data in a subsequent publication along with AUC SV measurements. It has also been observed that mAbs deviate from linear viscosity behavior above approximately 50 mg/ml. (The Shire group has investigated the impact of association on viscosity and the problems it causes for drug delivery by injection (Liu et al. 2005; Yadav et al. 2011b, 2012).) We have confirmed these nonlinear viscosity observations and furthermore observe similar deviations in *s*_w_ vs concentration plots. This has implications for SEDANAL analysis and requires a second order term, *k*_*s*2_*c*^2^, in the *s*/*s*_o_ and *D*/*D*_o_ phenomenological Eqs. (3, 4). This and a higher order 3rd virial coefficient, CM_1_, were previously incorporated into SEDANAL. The polymer field refers to this nonlinear deviation as clustering or low energy attraction (~ 5 kT or 3 kcal/mol) near the boundary for liquid–liquid phase separation (Fiore et al. 2018). Clustering is clearly a distinct phenomena to the self-association we describe here.

## Electronic supplementary material

Below is the link to the electronic supplementary material.

Supplementary file1 (DOCX 1635 kb)

Supplementary file2 (PDF 1715 kb)

Supplementary file3 (JPG 920 kb)

Supplementary file4 (JPG 950 kb)

Supplementary file5 (JPG 705 kb)

Supplementary file6 (JPG 553 kb)

Supplementary file7 (JPG 1017 kb)

Supplementary file8 (JPG 1628 kb)

Supplementary file9 (JPG 1307 kb)

Supplementary file10 (JPG 1319 kb)

Supplementary file11 (JPG 155 kb)

Supplementary file12 (JPG 255 kb)

Supplementary file13 (JPG 1315 kb)

Supplementary file14 (JPG 1487 kb)

Supplementary file15 (JPG 1294 kb)

Supplementary file16 (JPG 1467 kb)

Supplementary file17 (JPG 1721 kb)

Supplementary file18 (JPG 1630 kb)
