# Supplementary material for: Analysis of nonideality: insights from high concentration simulations of sedimentation velocity data
Source: Eur Biophys J. 2020 Nov 6;49(8):687–700. doi: 10.1007/s00249-020-01474-5 (PMC7701085; doi:10.1007/s00249-020-01474-5)

Supplemental Tables and Figures

Analysis of Nonideality: Insights from High Concentration Simulations of Sedimentation Velocity Data

J. J. Correia[1], R.T. Wright[2], P. J. Sherwood[3], W.F. Stafford[4]

[1] Dept. of Cell & Molecular Biology, Univ. of MS Medical Center, Jackson, MS
[2]Biophysics Group, Janssen Biotherapeutics, Spring House, PA
[3]Interactive Technology, Oakland, CA
[4]Department of Systems Biology, Harvard Medical School, Boston, MA

Methods. The hydrodynamic k_s and thermodynamic nonideality BM_1 for heterogeneous systems are provided as matrices, with elements, k_{ij} and $B_{ij}M_i$, representing self- and cross-term interactions (Table S1 and S2; Correia and Stafford 2015; Wright et al. 2018a). For example, an AA_2BC model involved four species (a monomer, a reversible dimer, an aggregated dimer and an aggregated trimer) where each sedimentation coefficient is a function of each concentration (equations S1-S4) in a time and radial position dependent manner (Figures S2AB). This defines a 4x4 matrix (Table 1) where the ith row, reading across, reflects the terms in the denominator for each s_i species. The coefficients k_{ij} in that row match c_j terms and reflect the concentration dependence due to species j on species i or k_{ij}*c_j. Alternatively, the jth column, reading down, reflects the influence of species j on the species i. For example, the first column reflects the influence of monomers on monomers, $k_{11}c_1$, monomers on reversal dimers, k_{21},c_1, monomers on aggregated dimers, $k_{31}c_1$, and monomers on aggregated trimers, $k_{41}c_1$. Thus, the diagonal represents the self-nonideality and the off diagonal terms represent the cross-term nonideality. The terms in a column are assumed to be constant reflecting the same backflow from species i. Attempts to measure k_{ij} cross terms involves binary mixtures where one component is titrated against another (Correia et al. 2016; Wright et al. 2018a). This is difficult if not impossible with aggregates and reversible complexes. The assumption is that nonideality is constant on a weight basis, although estimates of f/f_o allow alterations in specific k_{ij} terms (Rowe 1977).

$$s_1 = \frac{s_1^0}{1+k_{11}c_1+k_{12}c_2+k_{13}c_3 + k_{14}c_4} \qquad \text{S1}$$

$$s_2 = \frac{s_1^0}{1+k_{21}c_1+k_{22}c_2+k_{23}c_3 + k_{24}c_4} \qquad \text{S2}$$

$$s_3 = \frac{s_1^0}{1+k_{31}c_1+k_{32}c_2+k_{33}c_3 + k_{34}c_4} \qquad \text{S3}$$

$$s_4 = \frac{s_1^0}{1+k_{41}c_1+k_{42}c_2+k_{43}c_3 + k_{44}c_4} \qquad \text{S4}$$

SEDANAL models that include hydrodynamic k_s and thermodynamic nonideality BM_1 allow three fitting options: fit, hold, or matrix. The fit option allows a single value to be varied in a NLLS sense, but that value applies to all column elements in the implied matrix. Matrix allows the user to enter different values for each ij element in the matrix. The hold option allows a constant value to be used during the fit or simulation. This corresponds to the k_s and BM_1 items in the first column A in Figure 1, indicated by the blue color. The yellow colors indicate that values are derived or copied from the blue box to their left. Currently matrix elements cannot be fit individually due to 1) the reasonable assumption that backflow for each species is constant, and 2) that they are underdetermined. Future versions of SEDANAL will cautiously allow fitting of select matrix elements. Similar considerations apply to the $B_{ij}M_j$ matrix, Table S2.

Table S1
Hydrodynamic nonideality matrix of k_{ij} values in units of ml/mg.

		A	A_2	B	C
		k_{i1}	k_{i2}	k_{i3}	k_{i4}
A	k_{1j}	0.01	0.01	0.01	0.01
A_2	k_{2j}	0.01	0.01	0.01	0.01
B	k_{3j}	0.01	0.01	0.01	0.01
C	k_{4j}	0.01	0.01	0.01	0.01

Table S2
Thermodynamic nonideality matrix of $B_{ij}M_i$ values in units of ml/mg.

		A	A_2	B	C
		$B_{i1}M_1$	$B_{i2}M_2$	$B_{i3}M_3$	$B_{i4}M_4$
A	$B_{1j}M_j$	0.01	0.01	0.01	0.01
A_2	$B_{2j}M_j$	0.01	0.01	0.01	0.01
B	$B_{3j}M_j$	0.01	0.01	0.01	0.01
C	$B_{4j}M_j$	0.01	0.01	0.01	0.01

Figure S1 Asymptotic limitation on the ratio D/D_o as a function realistic positive values of k_s and BM_1 and total protein concentration (see equation 2; in the $\lim_{c\to\infty} = \frac{2BM_1c}{k_sc}$) A) $k_s=BM_1$; B) $BM_1 > k_s$ in a concentration dependent manner.

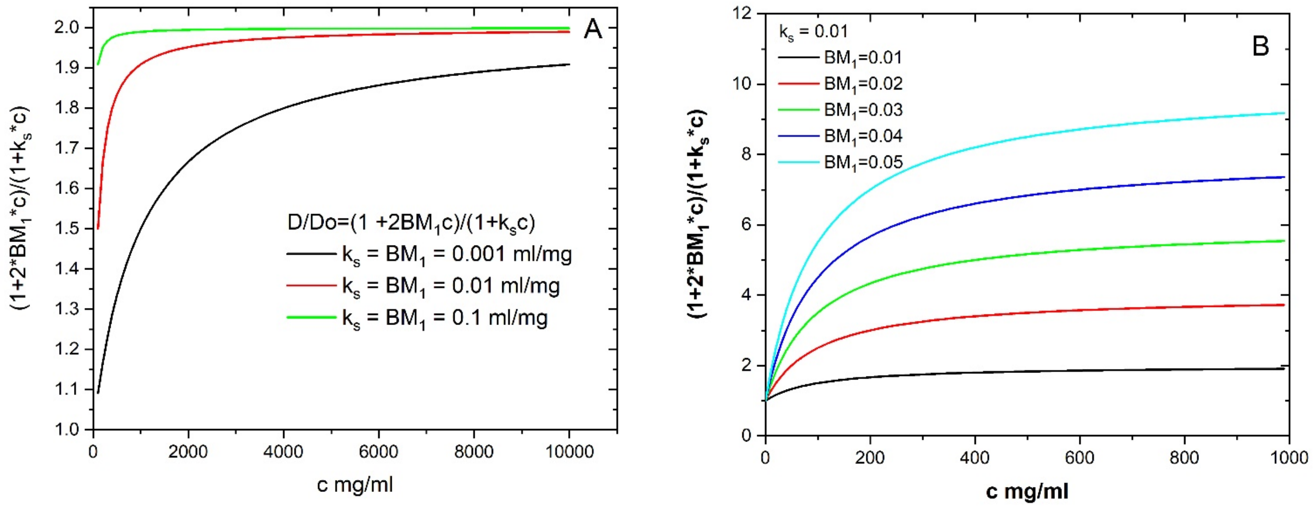

Figure S2 A) Plot of concentration vs radius for a monomer-dimer-trimer model for the 80th scan (SEDANAL output option) during a simulation with $k_s = BM_1$ = 10 ml/g and monomer concentration = 40 mg/ml. Note the JO effect in the monomer concentration distribution. B) The plot shows s/s_o and D/D_o for these data, as calculated using equations 3 and 4, plotted vs radius. This demonstrates the change in nonideality as a function of radial position and component concentrations. Claverie options under the advanced control button allows limitations on min s/s_o, max D/D_o and max concentration at the base.

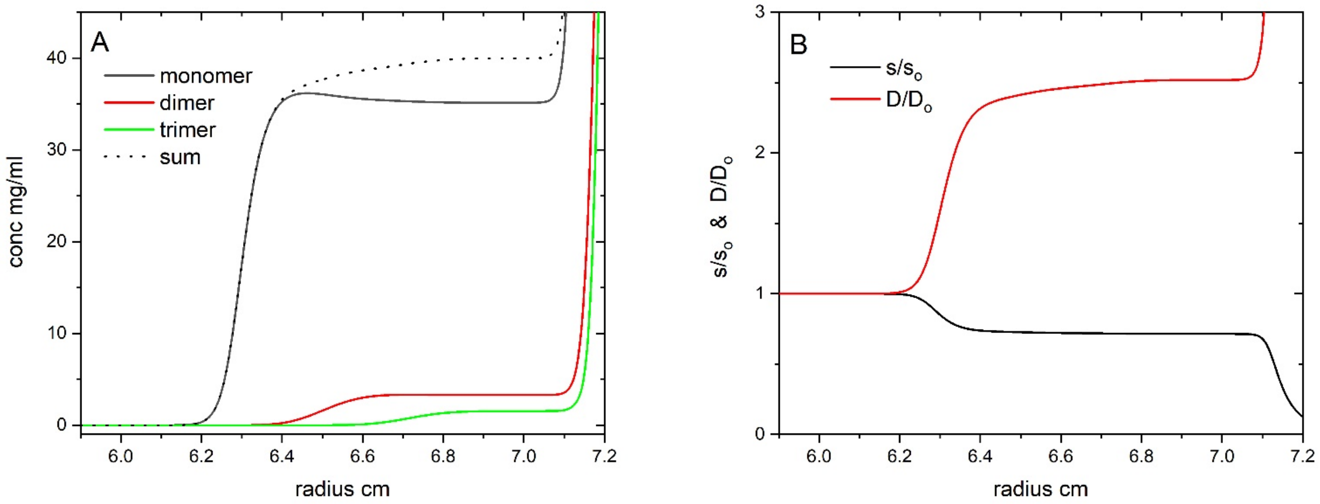

Figure S3 Wide Distribution (WD) Analysis (Stafford and Braswell, 2004) of an ABC model for 10 mg/ml Simponi and 10% dimeric (B/A) and 5% trimeric (C/A) aggregates. Panel A shows speed dependence and suggests that ABC or monomer-dimer-trimer species resolve better at higher speeds (larger σ, reduced molecular weight). Panel B shows WD analysis at 40K where different radial positions are averaged. This demonstrates data at high radial positions are preferred for best resolution. Radial positions greater than 6.5 cm clearly give the best separation and resolution for dimers and trimers. Data from 6.8 to 7.0 cm are typically used in the Figures 2-9, S4.

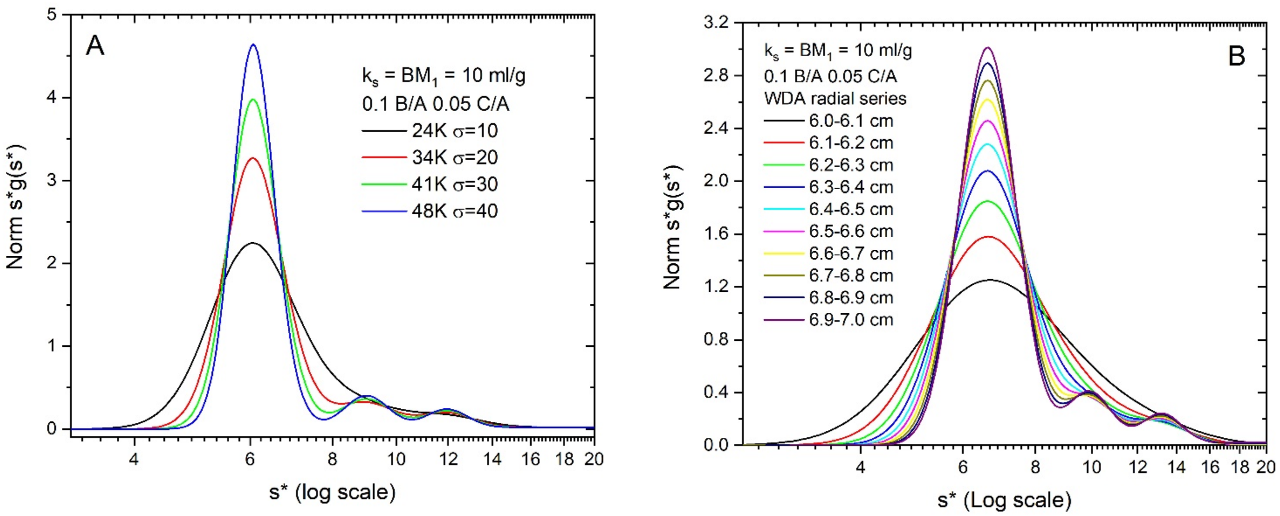

Figure S4 DCDT$^+$ and WD Analysis of an ABC model for 10 mg/ml Simponi and 10% dimeric and 5% trimeric aggregates. Panel A) shows DCDT$^+$ g(s*) vs s* plots while panel B) on the right shows WDA s*g(s*) vs log(s*) plots averaged from 6.8 to 7.0 cm. The use of large radial positions and the log scale in the WDA plot accentuates boundary sharpening and component separation.

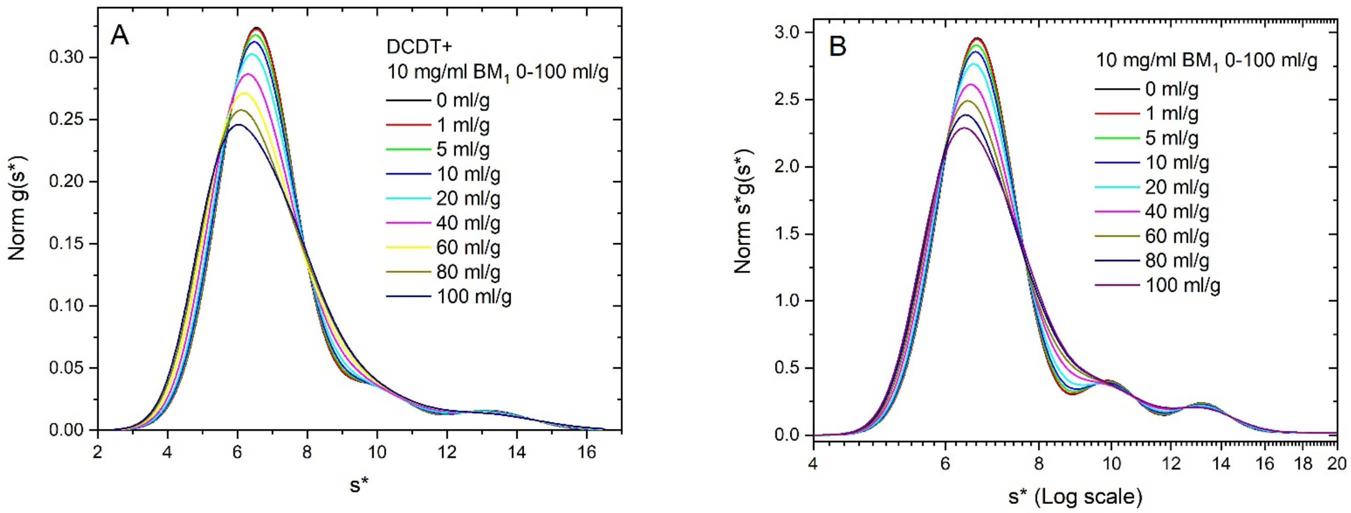

Figure S5 Screen dump of pre-processor images of Simponi simulation at 120 mg/ml with A) BM_1 = 0 ml/g and B) BM_1 = 10 ml/g. The y-axis is signal or absorbance while the x-axis is radial position in cm. Panel A) The BM_1 0 ml/g sample generates a pellet up to an absorbance of 38,000, or 27,142 mg/ml. Panel B) The BM_1 10 ml/g sample generates a pellet up to an absorbance or 2623, or 1873 mg/ml. This high concentration in the pellet spreads into the plateau region in a thermodynamically nonideal manner: at BM_1 = 0 ml/g the plateau is zero at 7.16 cm in the 400th scan; at BM_1 = 10 ml/g the plateau approaches zero at < 7.0 cm in the 400th scan. This behavior of an expanded back diffusion region is diagnostic of samples with large thermodynamic nonideality.

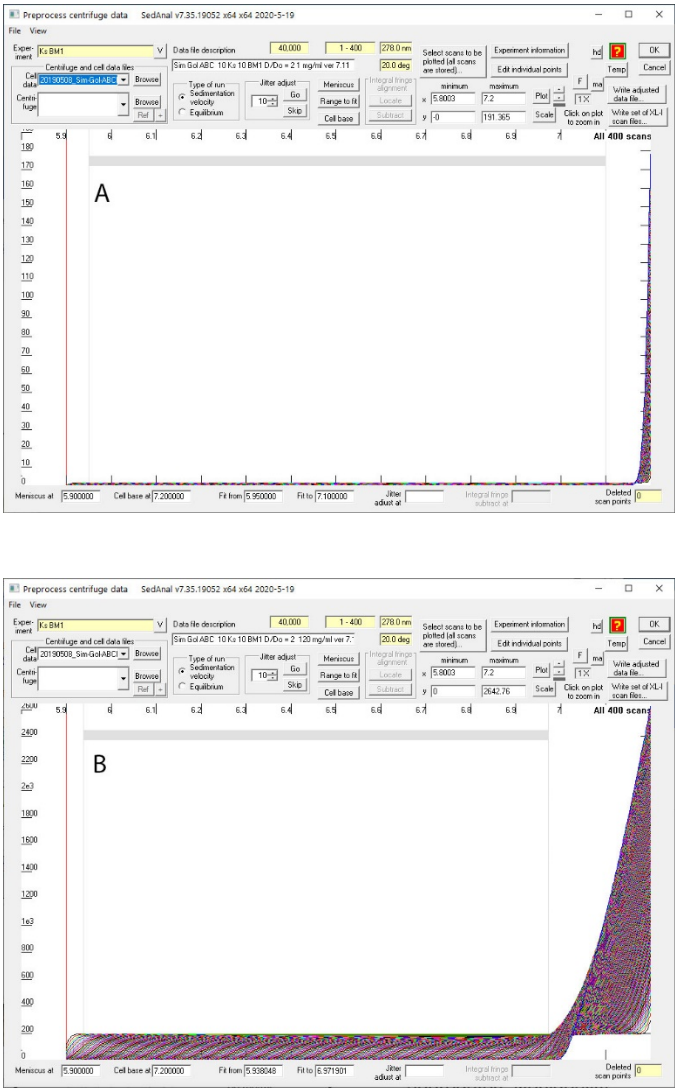

Figure S6 Panel A) DCDT$^+$ analysis, normalized g(s*) vs s*, of the concentration data presented in Figure 5. Panel B) is Figure 5 copied here for direct comparison. Analysis with g(s*) is typically presented vs s*. It is clear these data are not as well resolved at the data in Figure 5, panel B, especially the dimer and trimer aggregate peaks. The integrated results from these data are plotted as $1/s_w$ vs c in Figure 6.

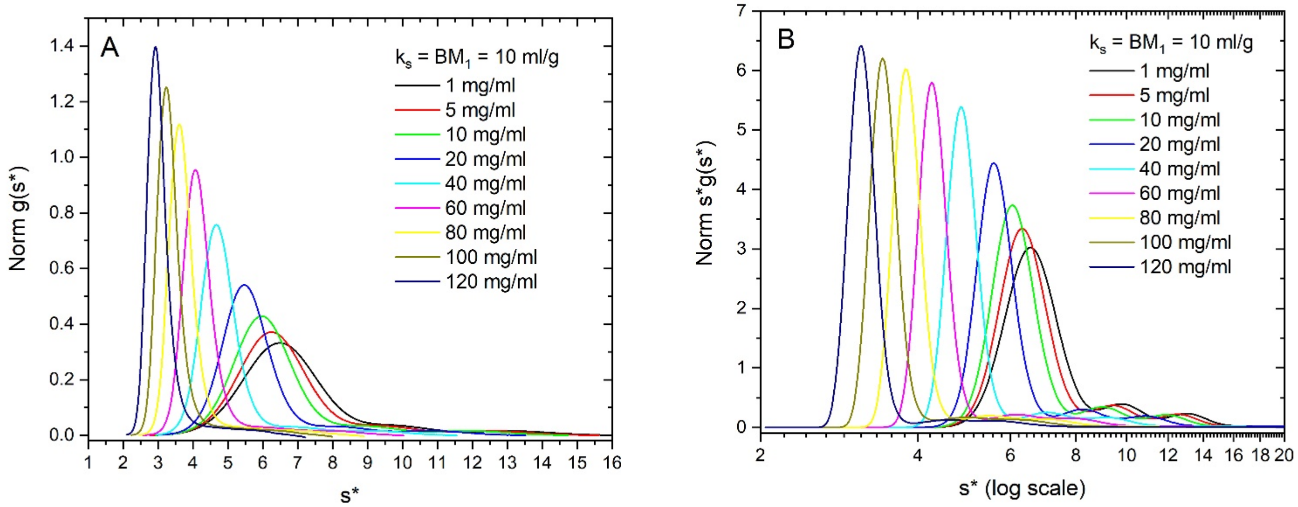

Figure S7 Panel A is DCDT^{+} analysis, normalized g(s*) vs s*, of the concentration data presented in Figure 8. Panel B is Figure 9 copied here for direct comparison. DCDT+ analysis for g(s*) is typically presented vs s*. It is clear these data are not as well resolved as the data in Figure 9, panel B, especially the dimer and trimer aggregate peaks. The integrated results from these data are plotted as $1/s_w$ vs c in Figure 10.

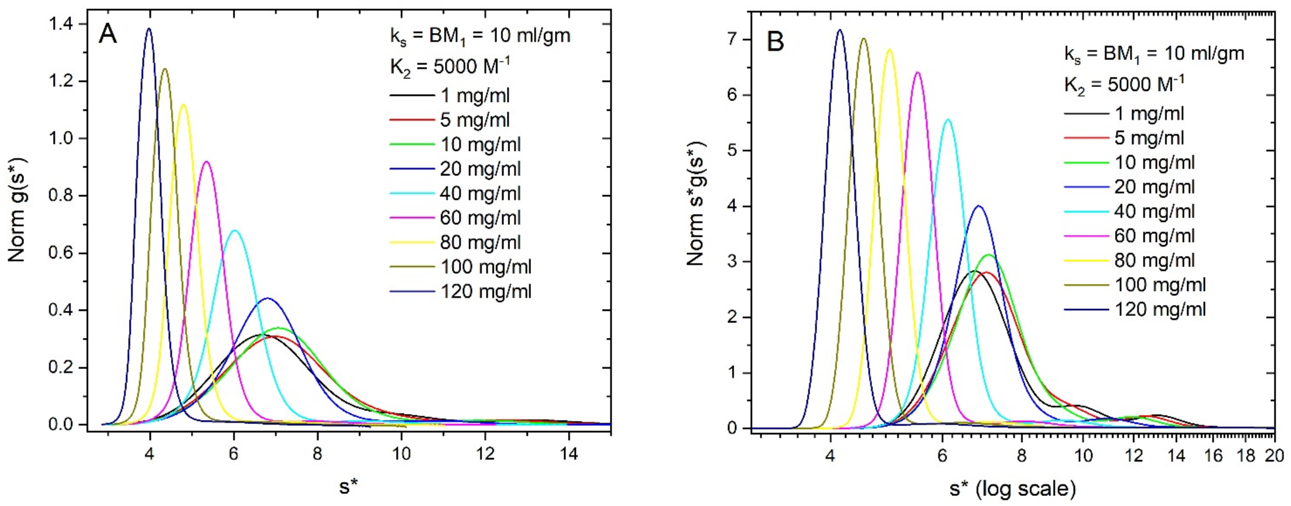

Figure S8 Panel A is a WDA plot of the ABCk$_s$BM$_1$ FDS data fit in Figure 7. Panel B is a WDA plot of the AA2BCk$_s$BM$_1$K$_2$ FDS data fit in Figure 11. The data is plotted in unnormalzed mode because the signal is the same in each sample in a tracer experiment.

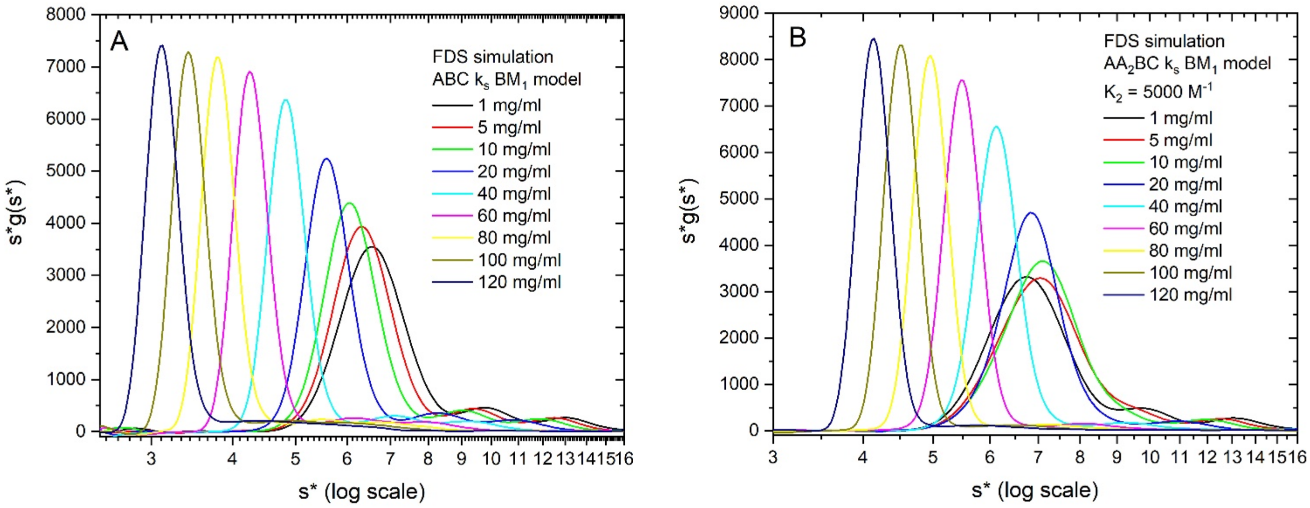

Supplement: Supplementary file 2 — Supplementary file2 (PDF 1715 kb) [file 249_2020_1474_MOESM2_ESM.pdf]
