# Supplementary figures and images for: Analysis of nonideality: insights from high concentration simulations of sedimentation velocity data
Source: Eur Biophys J. 2020 Nov 6;49(8):687–700. doi: 10.1007/s00249-020-01474-5 (PMC7701085; doi:10.1007/s00249-020-01474-5)

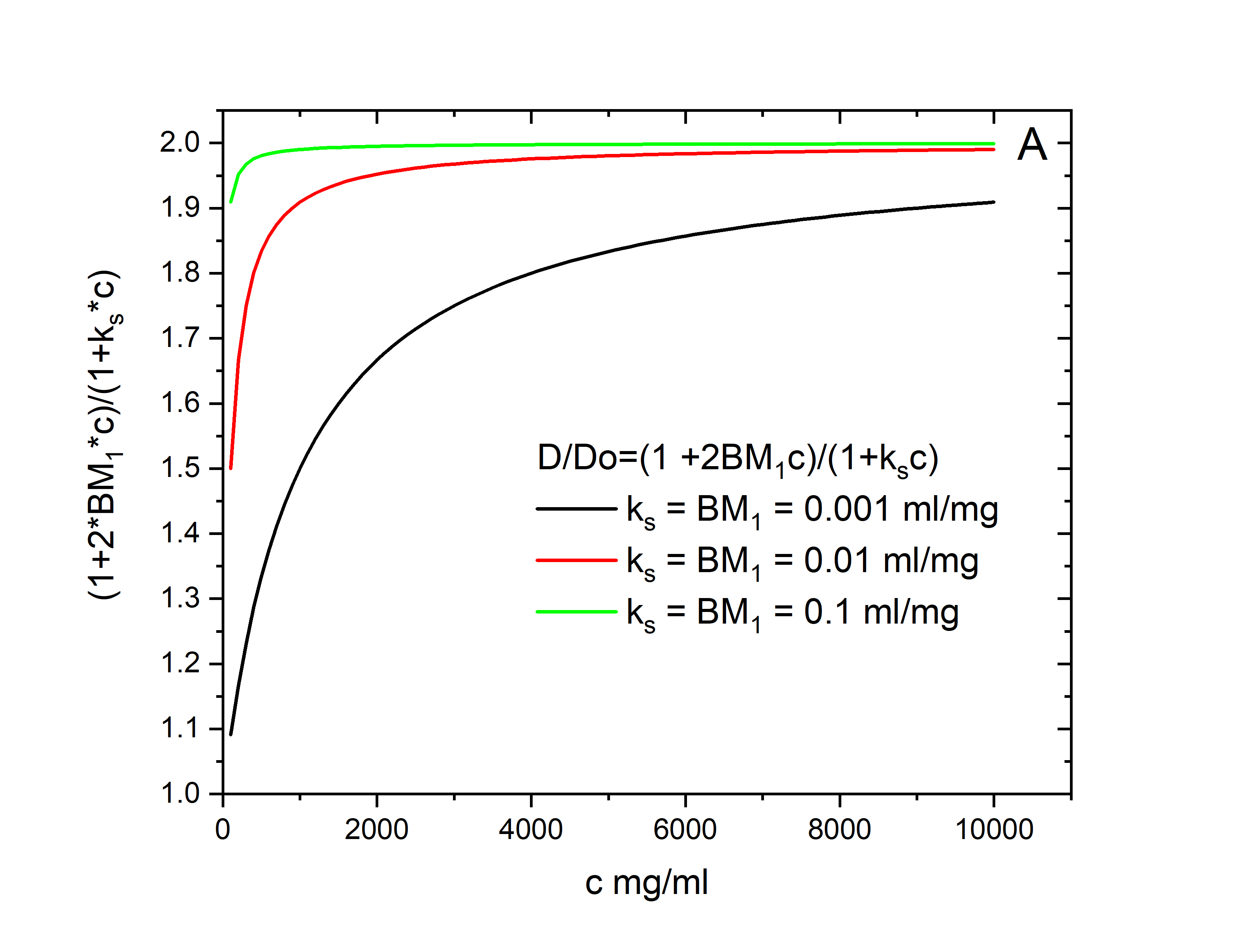

Supplement: Supplementary file 3 — Supplementary file3 (JPG 920 kb) [file 249_2020_1474_MOESM3_ESM.jpg]

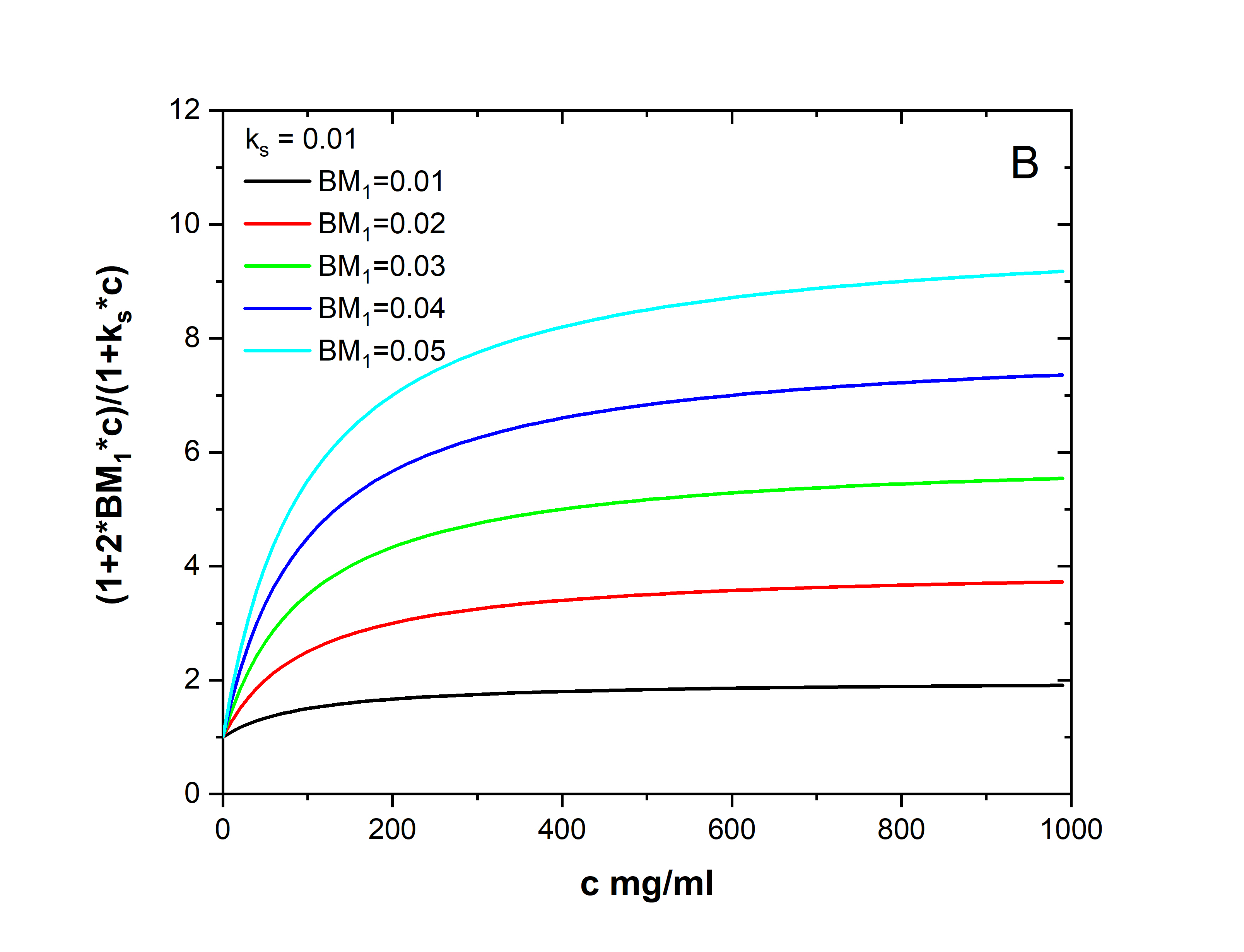

Supplement: Supplementary file 4 — Supplementary file4 (JPG 950 kb) [file 249_2020_1474_MOESM4_ESM.jpg]

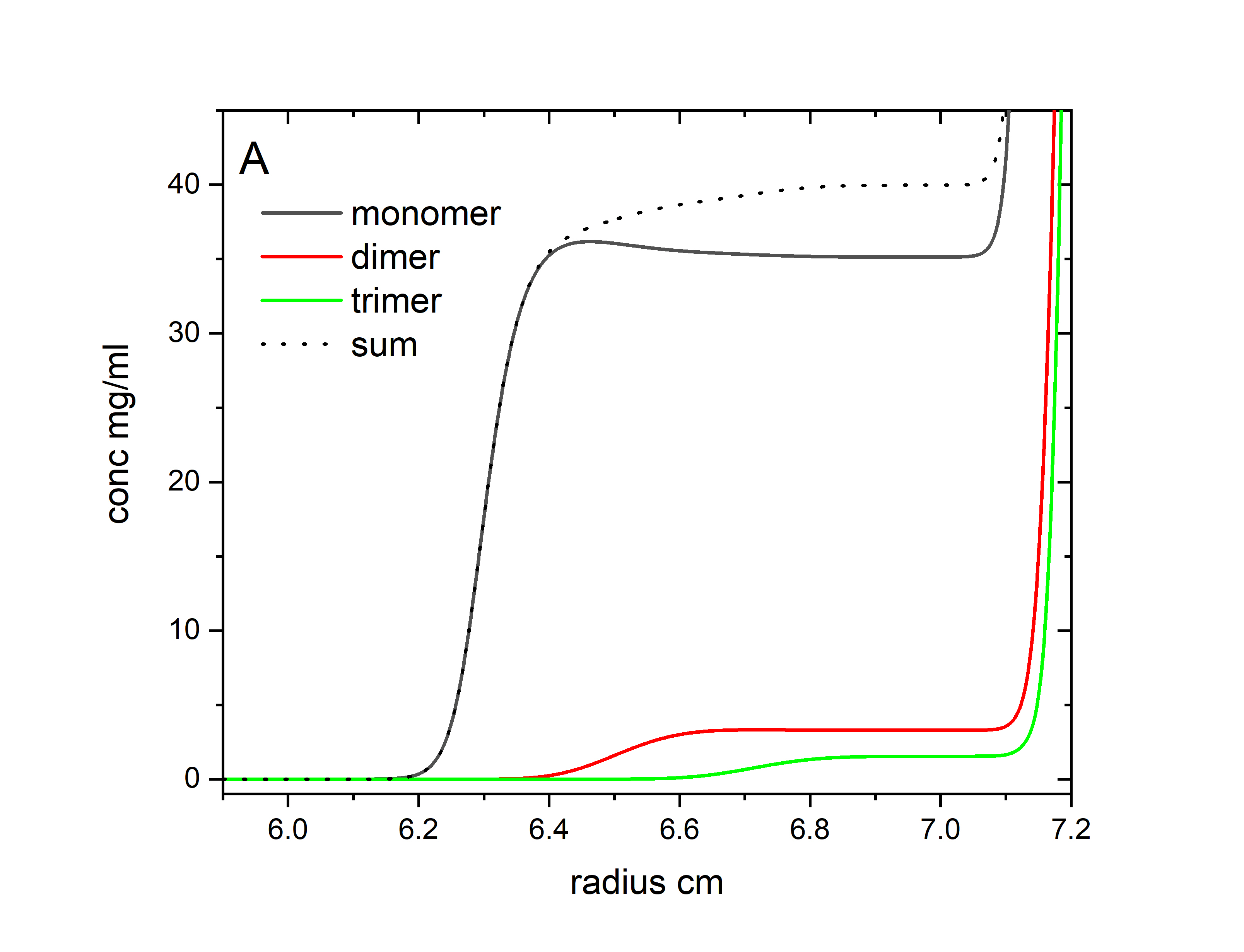

Supplement: Supplementary file 5 — Supplementary file5 (JPG 705 kb) [file 249_2020_1474_MOESM5_ESM.jpg]

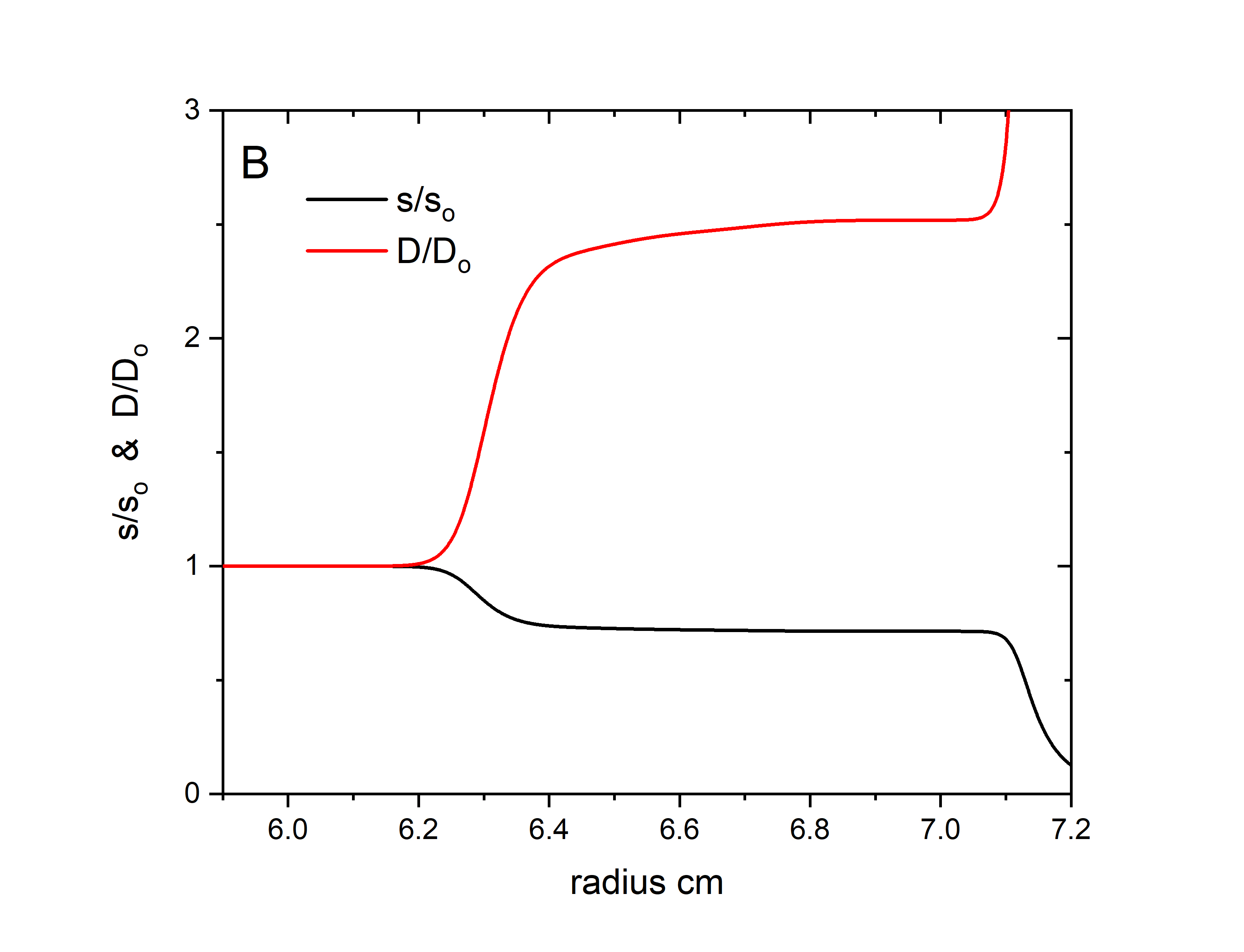

Supplement: Supplementary file 6 — Supplementary file6 (JPG 553 kb) [file 249_2020_1474_MOESM6_ESM.jpg]

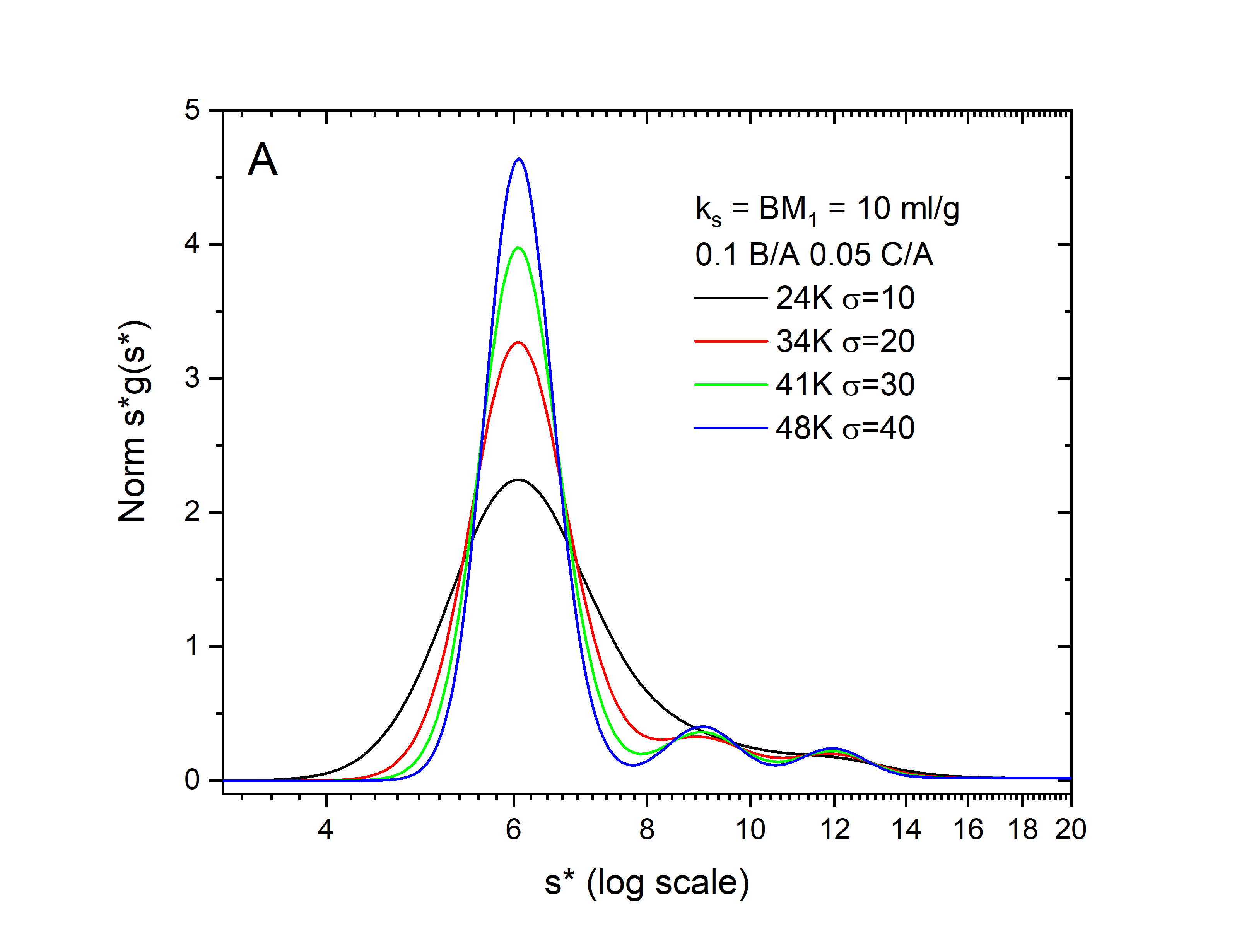

Supplement: Supplementary file 7 — Supplementary file7 (JPG 1017 kb) [file 249_2020_1474_MOESM7_ESM.jpg]

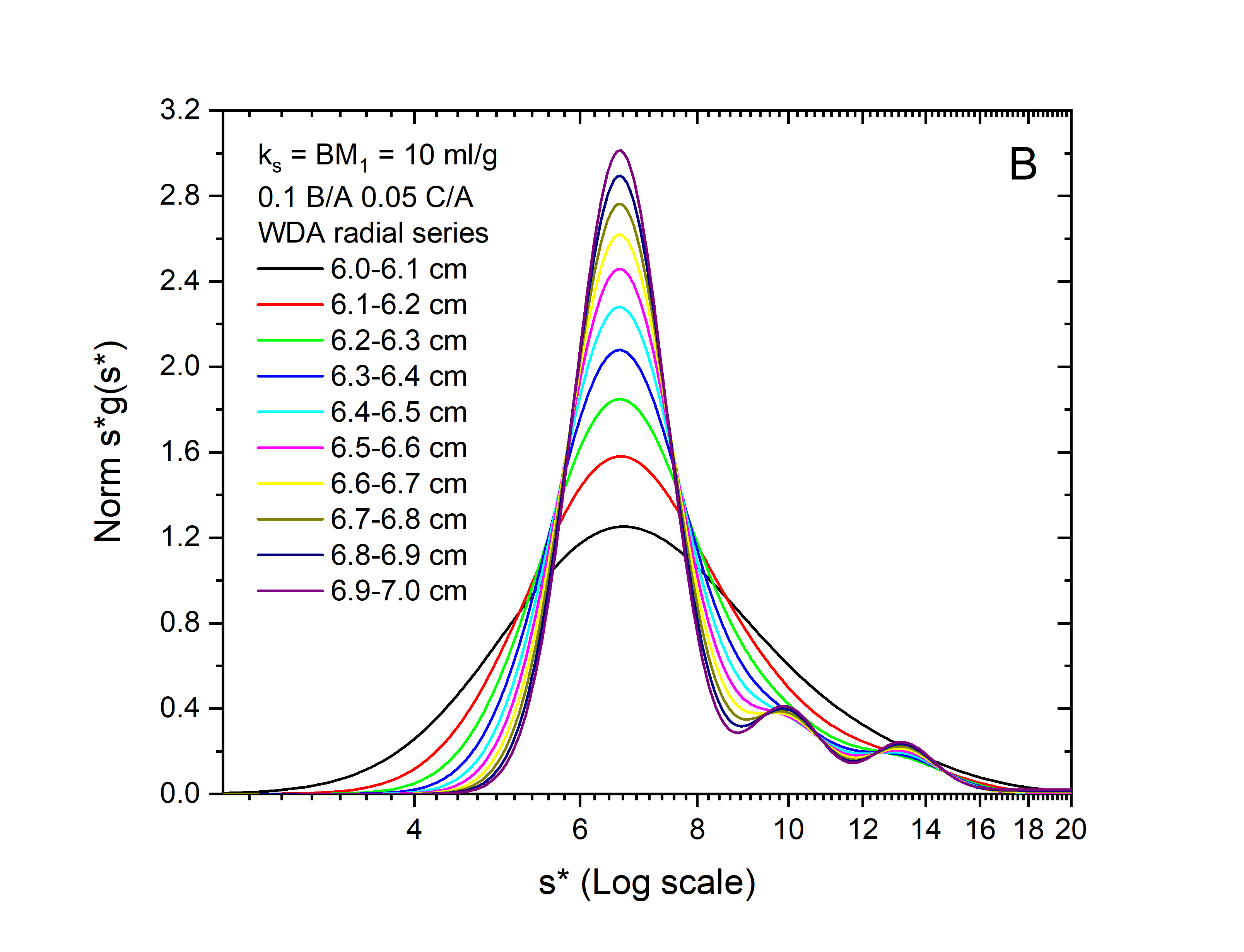

Supplement: Supplementary file 8 — Supplementary file8 (JPG 1628 kb) [file 249_2020_1474_MOESM8_ESM.jpg]

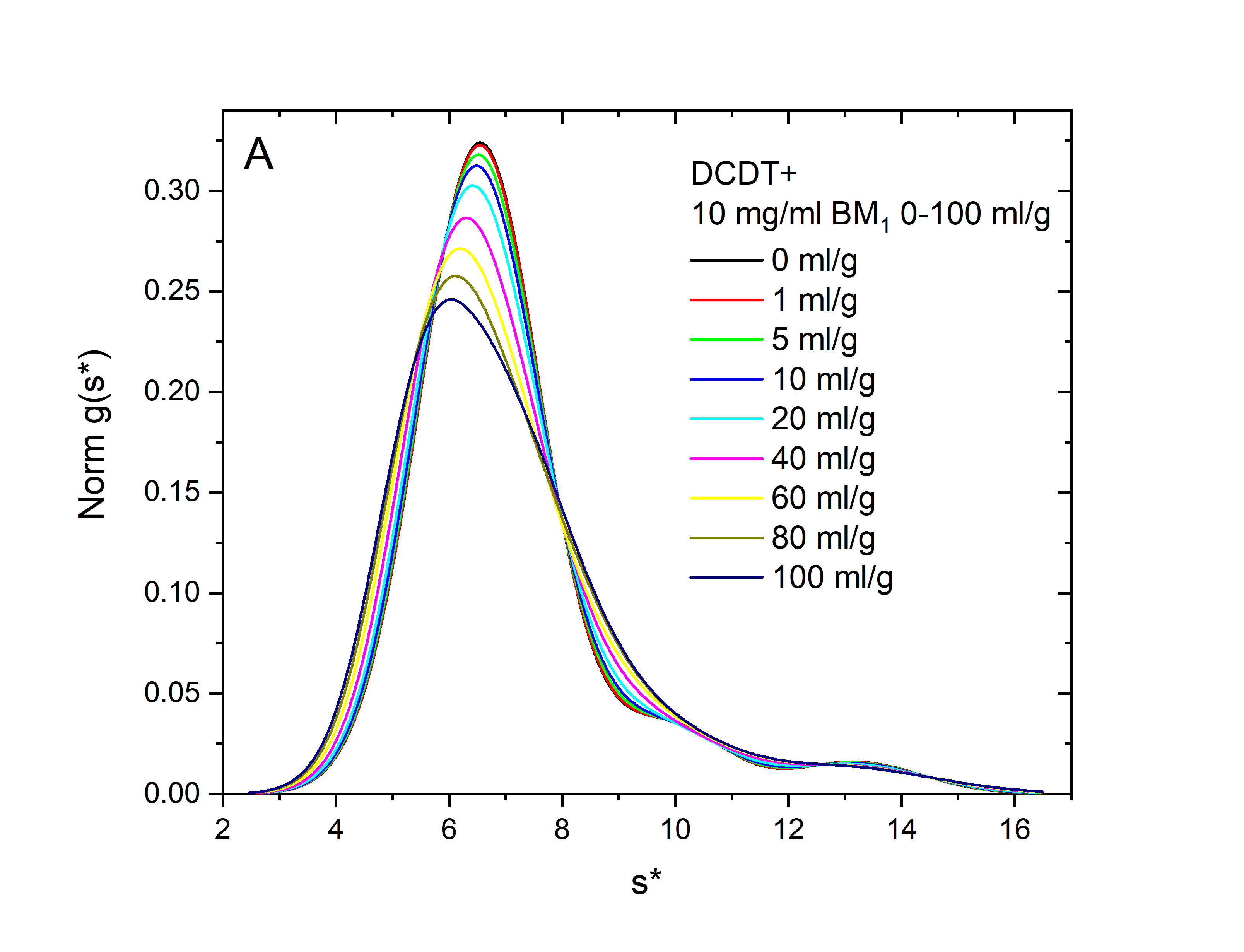

Supplement: Supplementary file 9 — Supplementary file9 (JPG 1307 kb) [file 249_2020_1474_MOESM9_ESM.jpg]

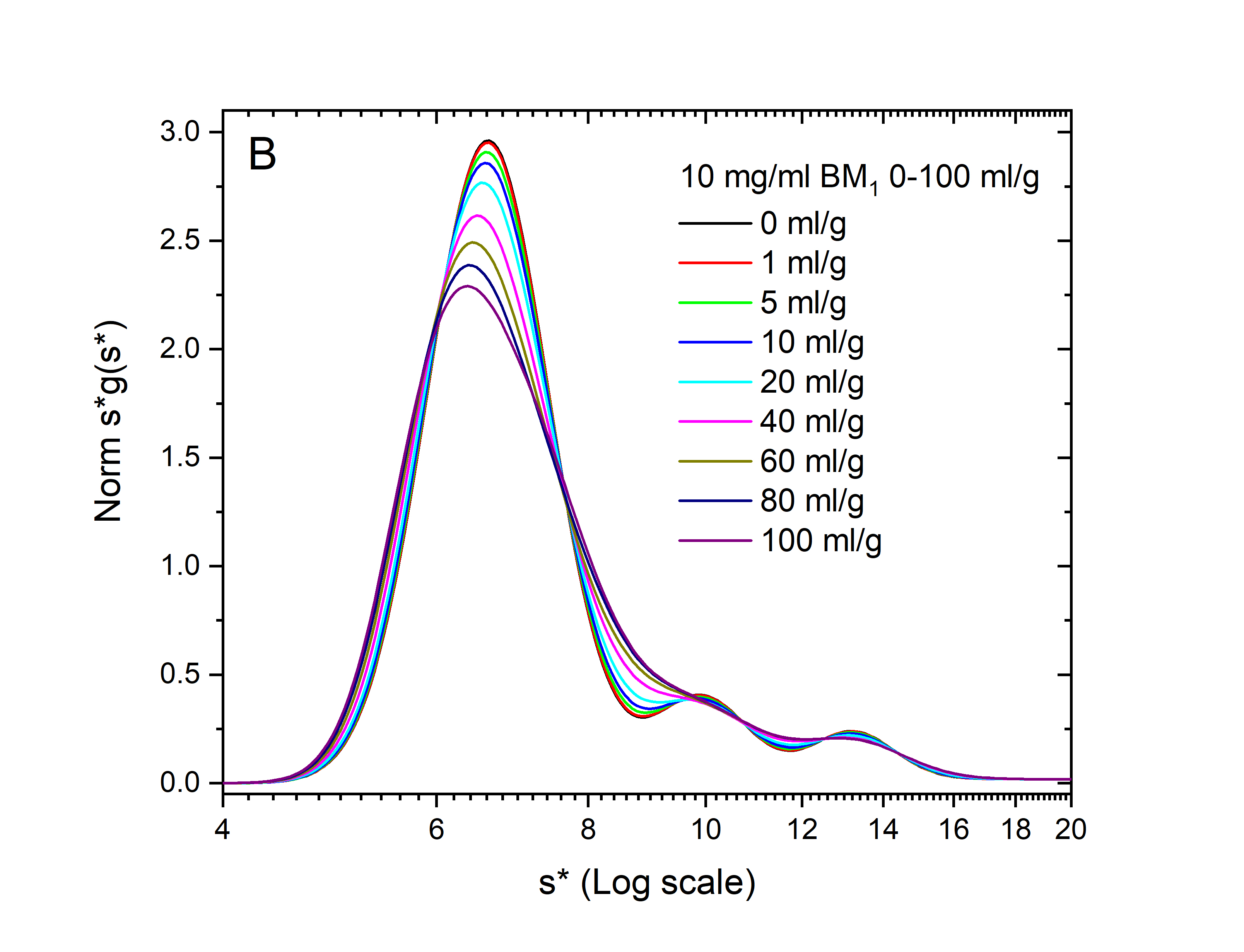

Supplement: Supplementary file 10 — Supplementary file10 (JPG 1319 kb) [file 249_2020_1474_MOESM10_ESM.jpg]

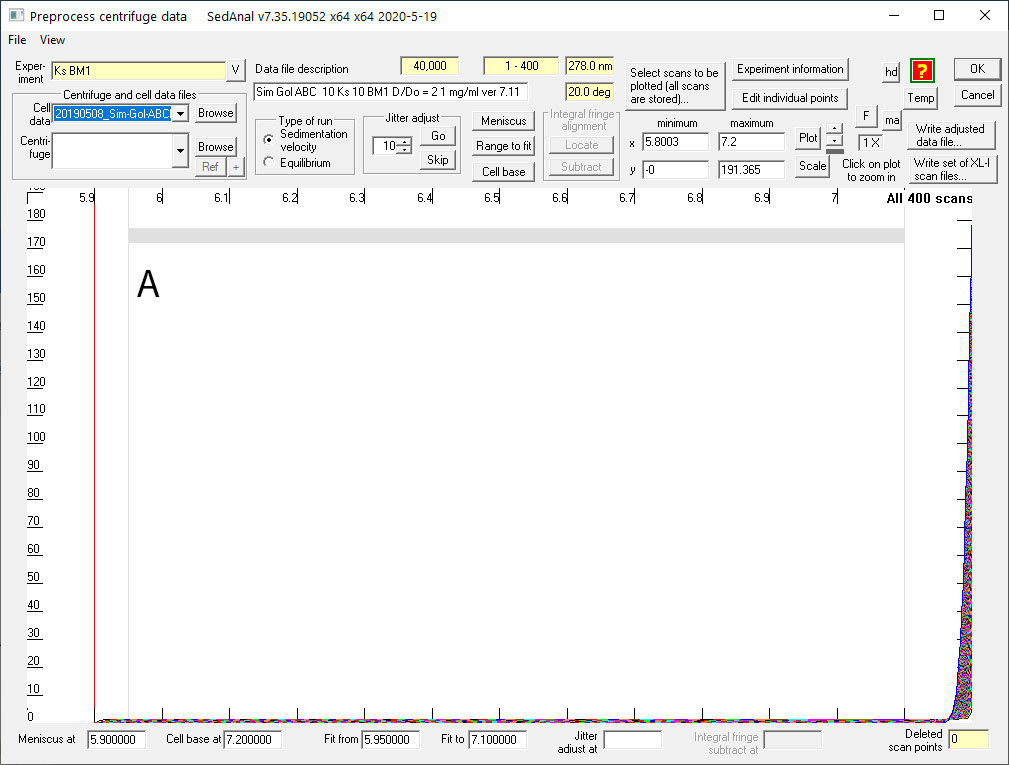

Supplement: Supplementary file 11 — Supplementary file11 (JPG 155 kb) [file 249_2020_1474_MOESM11_ESM.jpg]

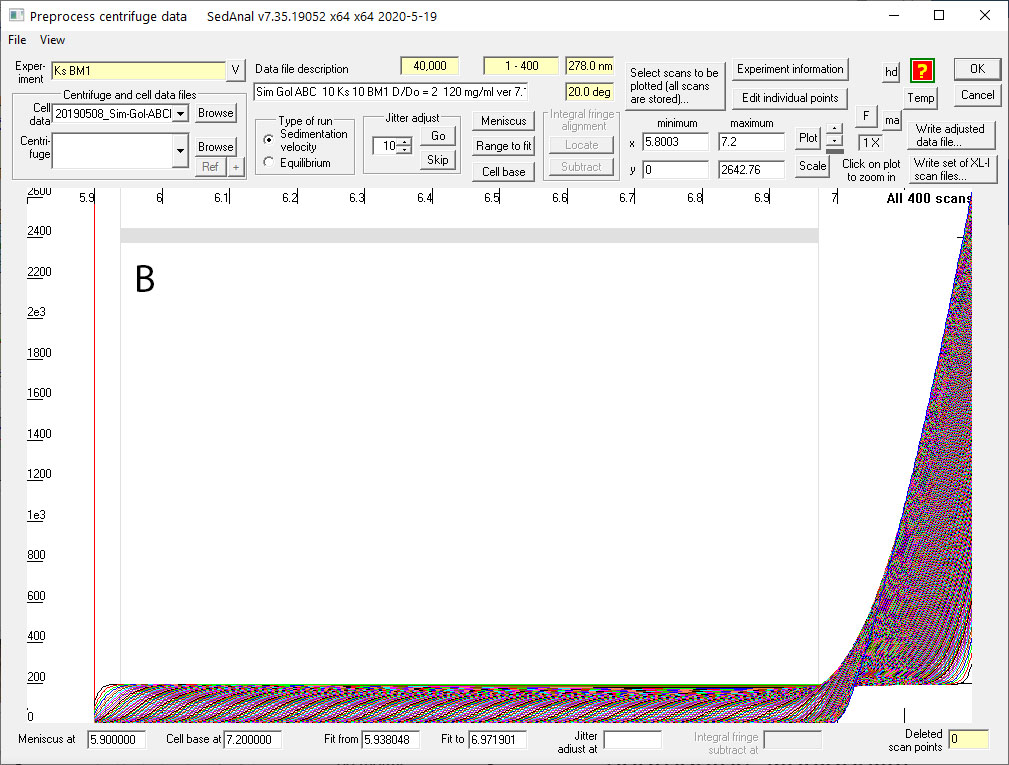

Supplement: Supplementary file 12 — Supplementary file12 (JPG 255 kb) [file 249_2020_1474_MOESM12_ESM.jpg]

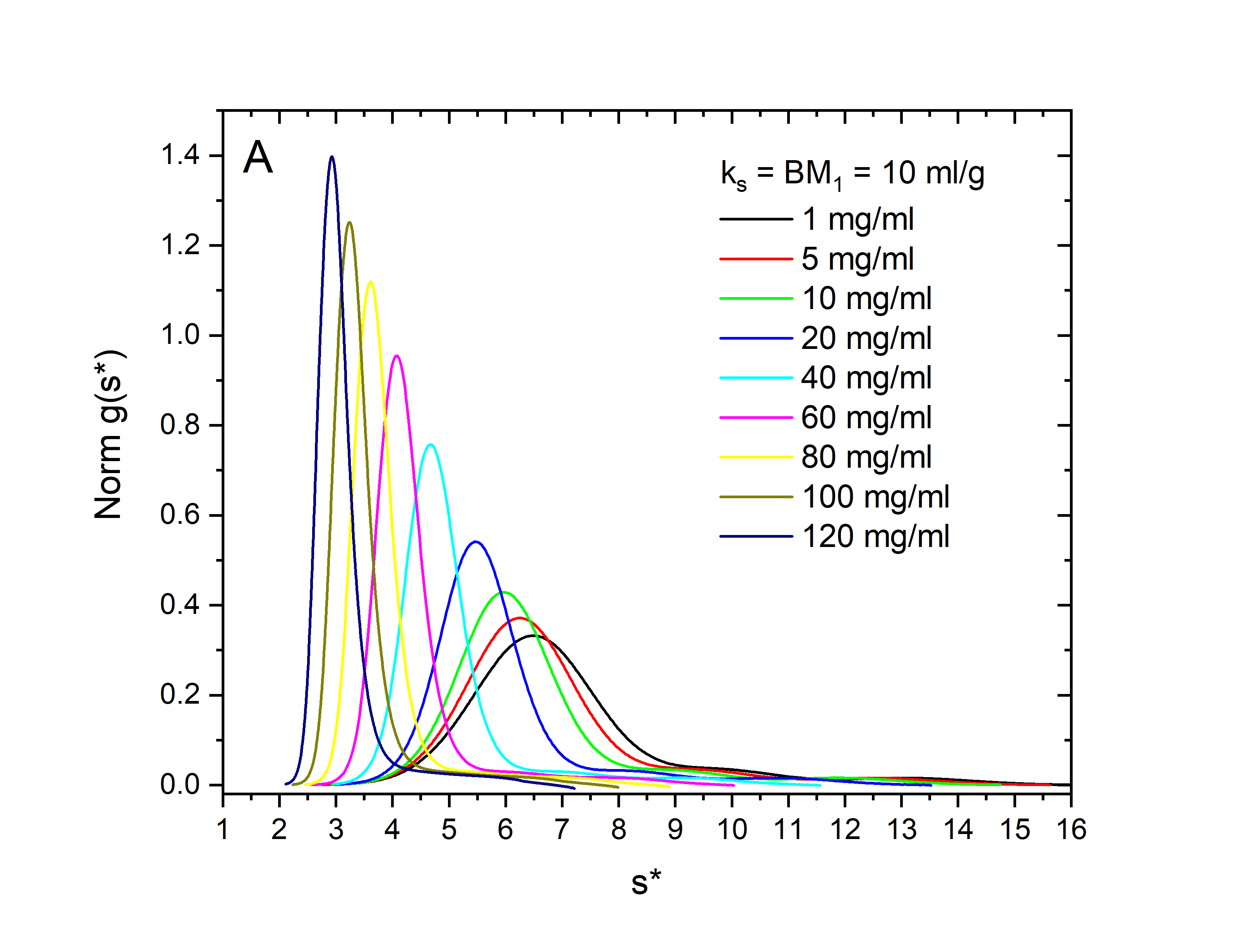

Supplement: Supplementary file 13 — Supplementary file13 (JPG 1315 kb) [file 249_2020_1474_MOESM13_ESM.jpg]

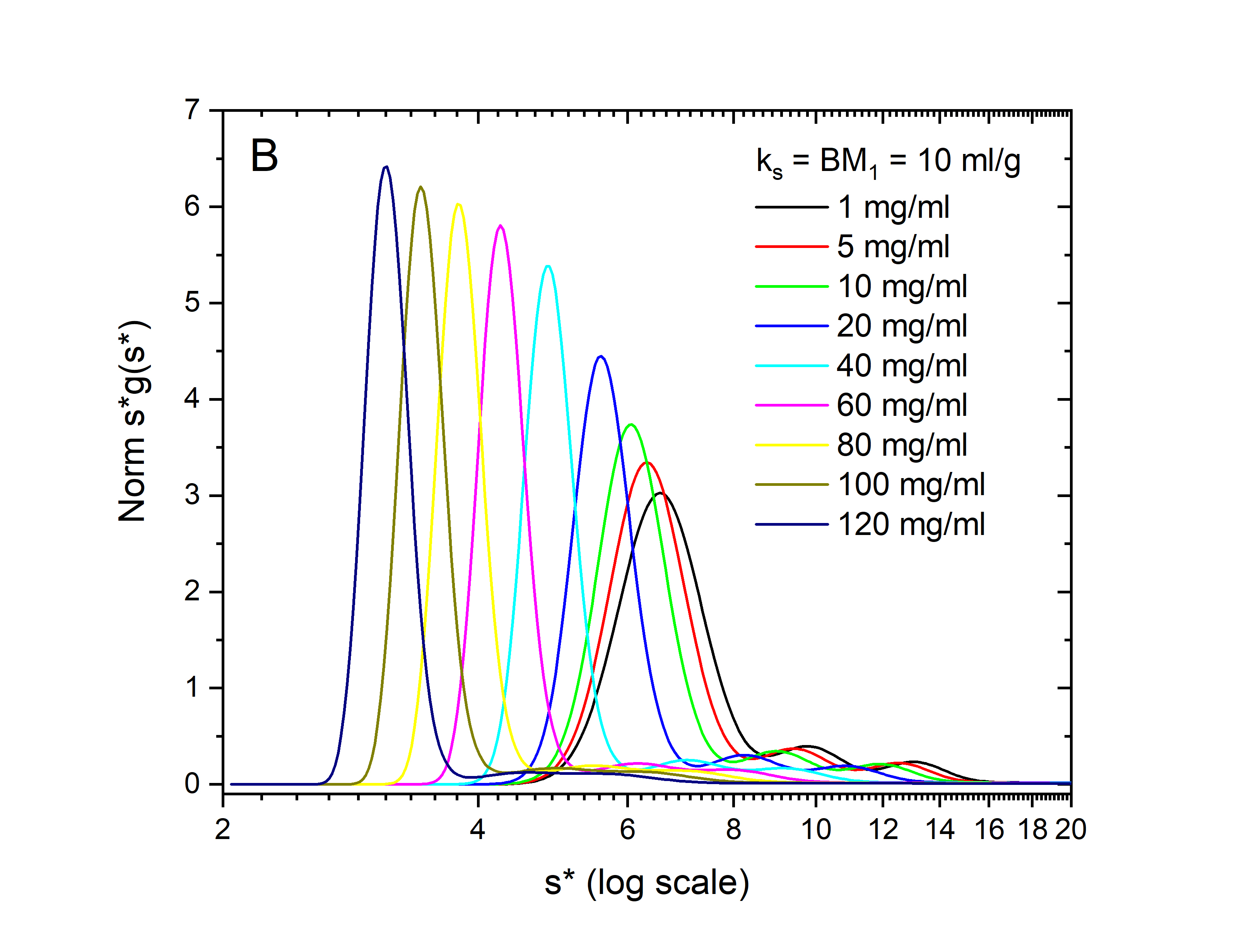

Supplement: Supplementary file 14 — Supplementary file14 (JPG 1487 kb) [file 249_2020_1474_MOESM14_ESM.jpg]

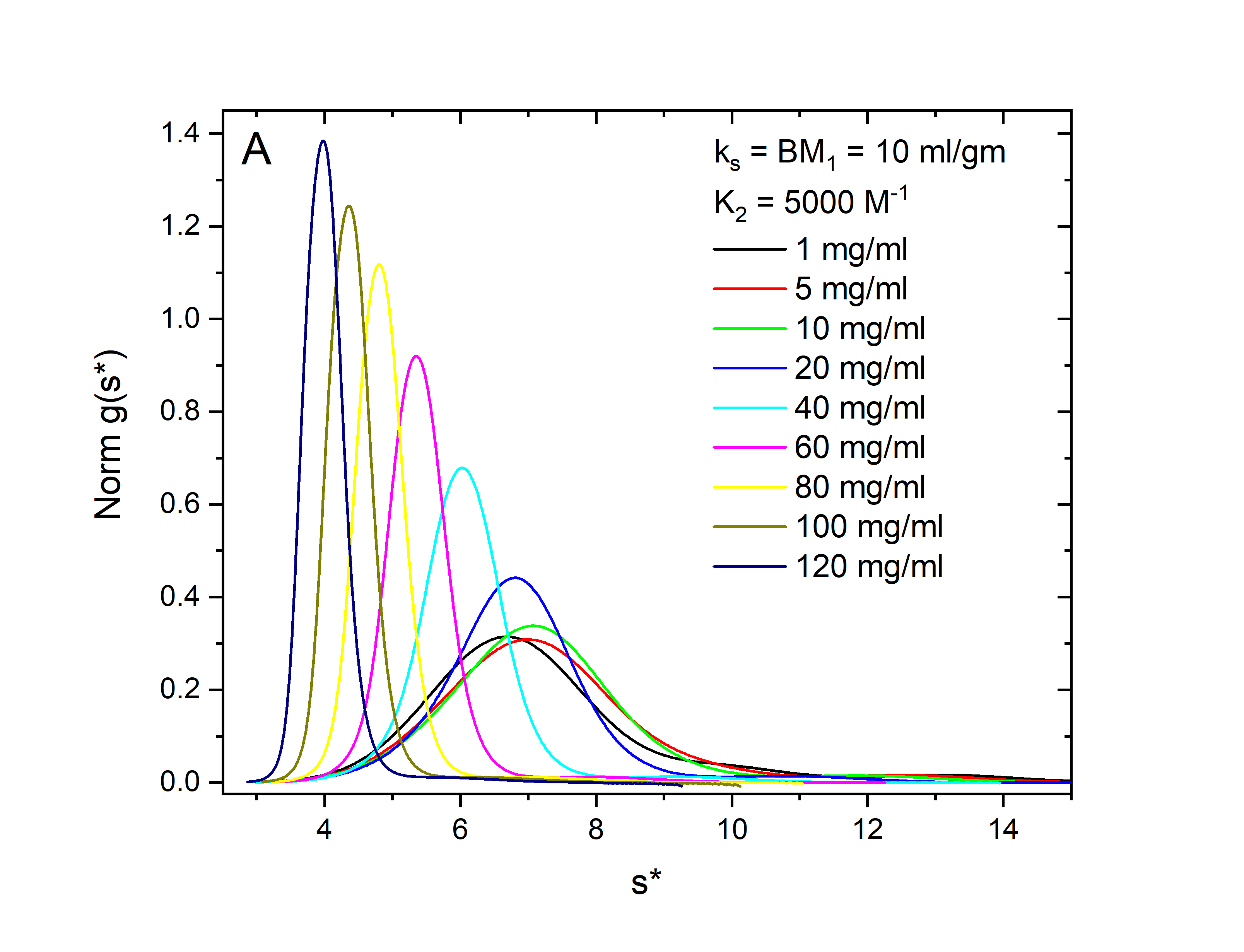

Supplement: Supplementary file 15 — Supplementary file15 (JPG 1294 kb) [file 249_2020_1474_MOESM15_ESM.jpg]

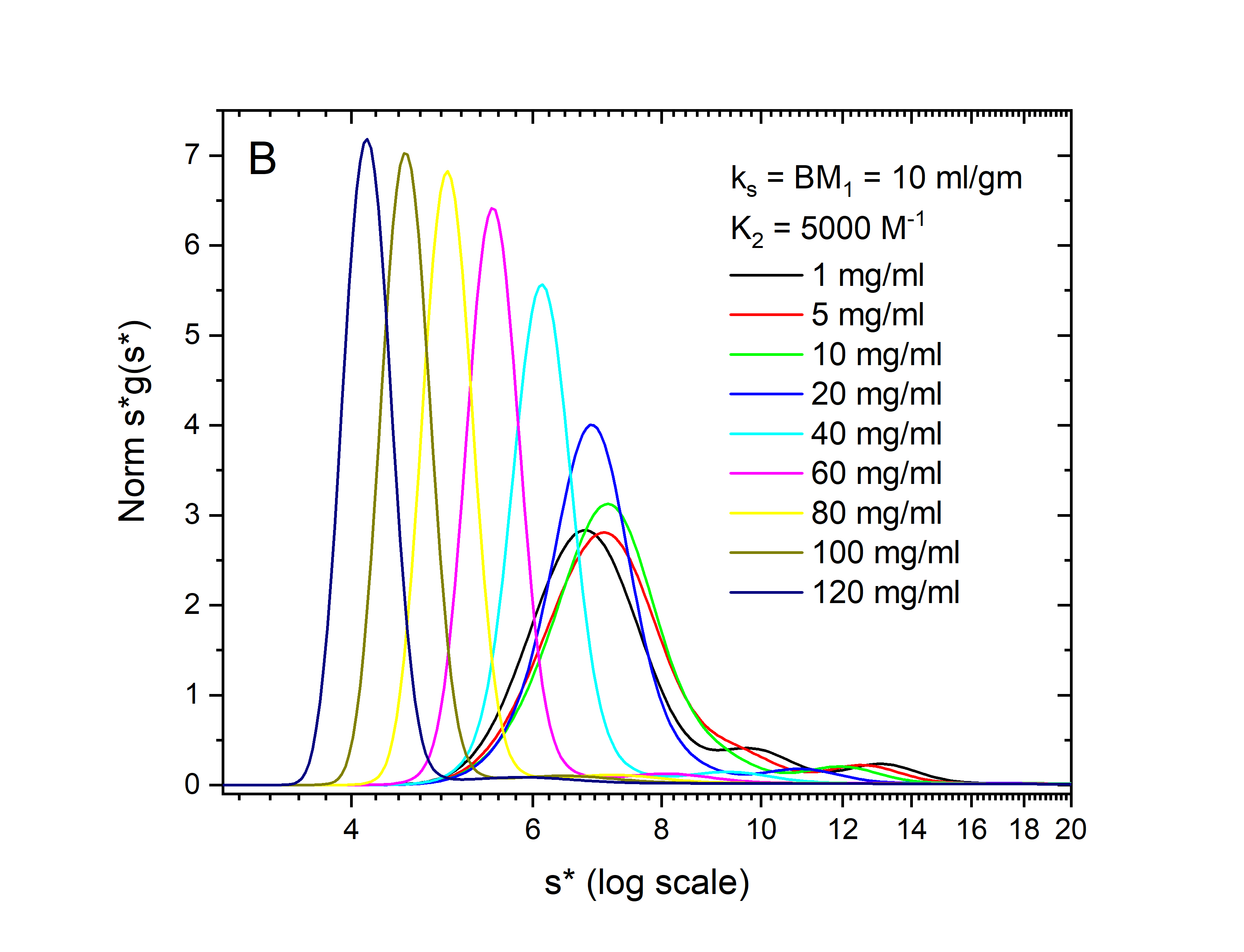

Supplement: Supplementary file 16 — Supplementary file16 (JPG 1467 kb) [file 249_2020_1474_MOESM16_ESM.jpg]

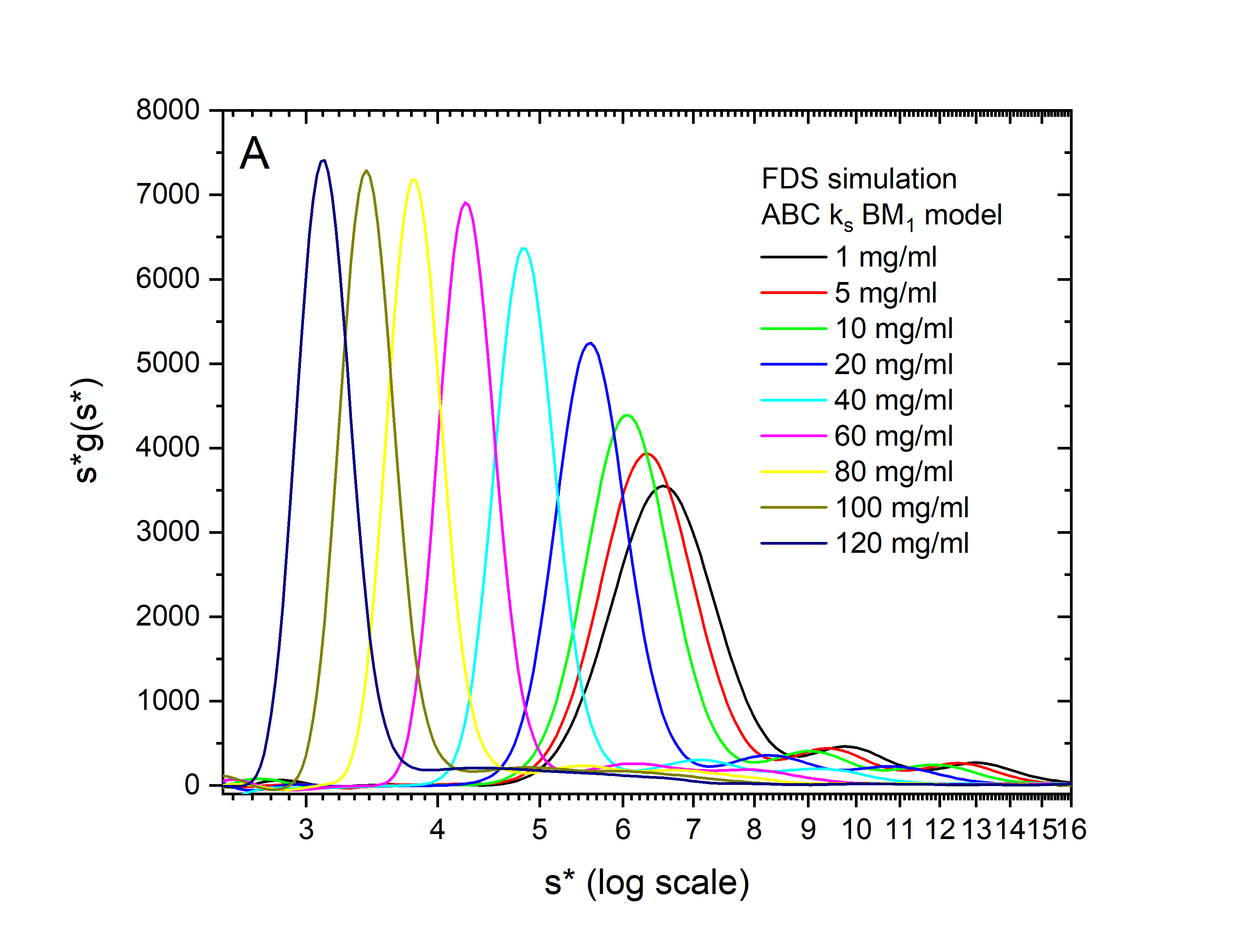

Supplement: Supplementary file 17 — Supplementary file17 (JPG 1721 kb) [file 249_2020_1474_MOESM17_ESM.jpg]

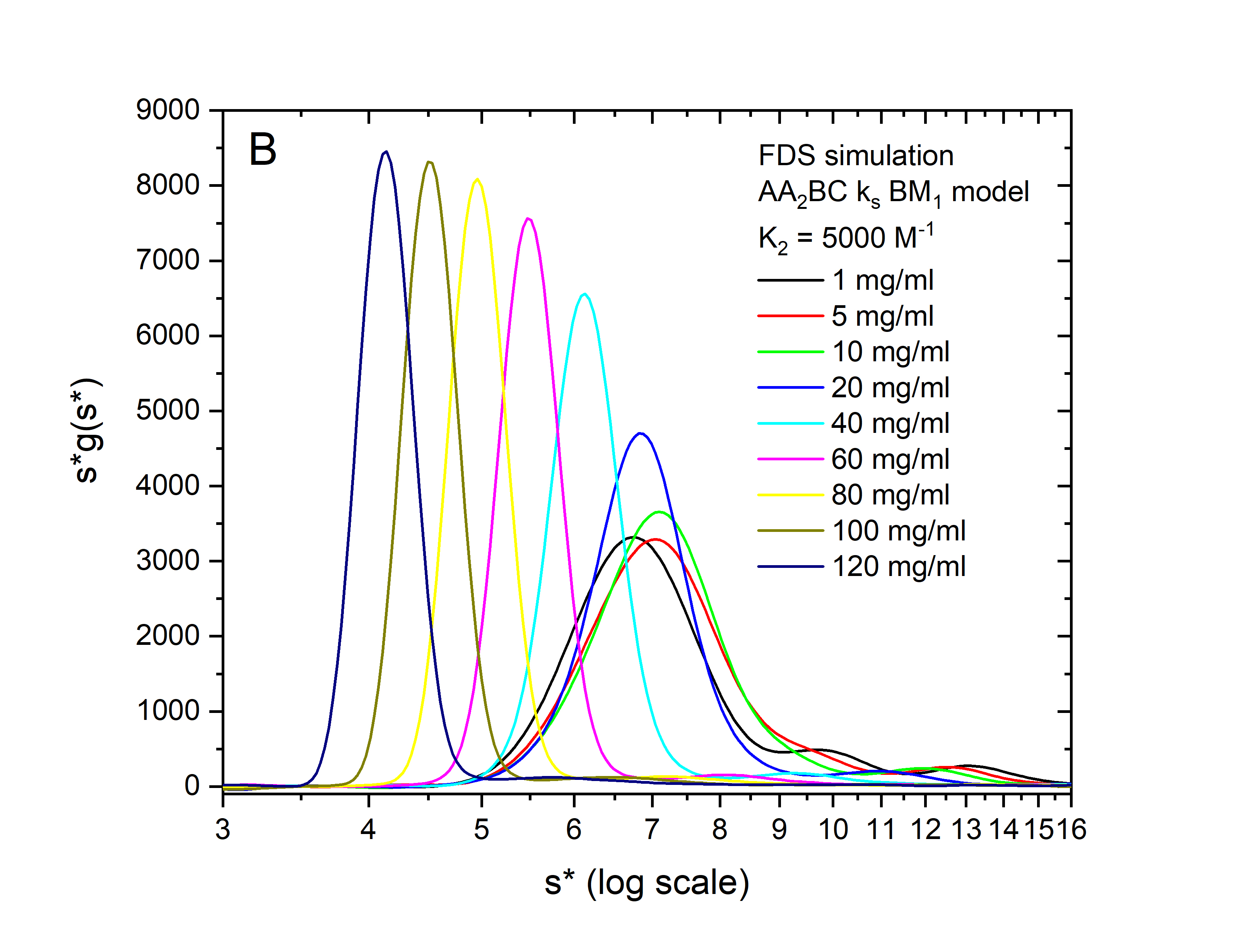

Supplement: Supplementary file 18 — Supplementary file18 (JPG 1630 kb) [file 249_2020_1474_MOESM18_ESM.jpg]
